# A Multinational Cluster Randomised Controlled Trial to Assess the Efficacy of ‘11+ Kids’: A Warm-Up Programme to Prevent Injuries in Children’s Football

**DOI:** 10.1007/s40279-017-0834-8

**Published:** 2017-12-22

**Authors:** Roland Rössler, Astrid Junge, Mario Bizzini, Evert Verhagen, Jiri Chomiak, Karen aus der Fünten, Tim Meyer, Jiri Dvorak, Eric Lichtenstein, Florian Beaudouin, Oliver Faude

**Affiliations:** 1 Department of Sport, Exercise and Health, University of Basel, Birsstrasse 320 B, 4052 Basel, Switzerland; 2 Amsterdam Collaboration on Health & Safety in Sports and Department of Public and Occupational Health, Amsterdam Movement Science, VU University Medical Center, Amsterdam, Netherlands; 3 Swiss Concussion Center, Zurich, Switzerland; 4 Schulthess Clinic, Zurich, Switzerland; 5 Medical School Hamburg, Hamburg, Germany; 6 Orthopaedic Department 1st Faculty of Medicine Charles University and IPVZ and Hospital Na Bulovce and FIFA med. Centre, Prague Czech Republic, Prague, Czech Republic; 7 Institute of Sports and Preventive Medicine, Saarland University, Saarbrücken, Germany

## Abstract

**Objective:**

The objective of this study was to assess the efficacy of a newly developed warm-up programme (‘11+ Kids’) regarding its potential to reduce injuries in children’s football.

**Methods:**

Children’s football teams (under 9 years, under 11 years, and under 13 years age groups) from Switzerland, Germany, the Czech Republic and the Netherlands were invited. Clubs were randomised to an intervention group and a control group, and followed for one season. The intervention group replaced their usual warm-up by ‘11+ Kids’, while the control group warmed up as usual. The primary outcome was the overall risk of football-related injuries. Secondary outcomes were the risks of severe and lower extremity injuries. We calculated hazard ratios using extended Cox models, and performed a compliance analysis.

**Results:**

In total, 292,749 h of football exposure of 3895 players were recorded. The mean age of players was 10.8 (standard deviation 1.4) years. During the study period, 374 (intervention group = 139; control group = 235) injuries occurred. The overall injury rate in the intervention group was reduced by 48% compared with the control group (hazard ratio 0.52; 95% confidence interval 0.32–0.86). Severe (74% reduction, hazard ratio 0.26; 95% confidence interval 0.10–0.64) and lower extremity injuries (55% reduction, hazard ratio 0.45; 95% confidence interval 0.24–0.84) were also reduced. Injury incidence decreased with increasing compliance.

**Conclusion:**

‘11+ Kids’ is efficacious in reducing injuries in children’s football. We observed considerable effects for overall, severe and lower extremity injuries. The programme should be performed at least once per week to profit from an injury preventive effect. However, two sessions per week can be recommended to further increase the protective benefit.

**Trial Registration:**

ClinicalTrials.gov identifier: NCT02222025.

**Electronic supplementary material:**

The online version of this article (10.1007/s40279-017-0834-8) contains supplementary material, which is available to authorized users.

## Key Points

**Table:** 

The newly developed injury prevention programme ‘11+ Kids’ is efficacious in reducing football injuries in children.
Considerable protective benefits were found for overall injuries, severe injuries and lower extremity injuries.
Injury incidence decreased with increasing compliance.

## Background

Worldwide, the majority of football players (58%) is younger than 18 years of age [1] and almost three quarters of these young players are under 14 years of age [2]. However, epidemiological data on football injuries in this age group are rare [3], and only one prospective large-scale study focused on injuries in children’s football [4, 5]. The characteristics of football injuries in 7- to 12-year-old children differ from youth and adult players. For example, the proportions of bone injuries and injuries to the upper extremities are higher in children than in older players [3, 4]. Thus, preventive programmes proven efficacious in late adolescent or adult players need to be adapted for younger age groups to accommodate for the specific injury profile and maturational status of children [3].

Several studies have investigated the exercise-based injury prevention programme ‘11+’ in players aged 14 years and older, and reported reductions (between 32 and 72%) in the incidence of all and/or lower extremity injuries [6–9]. Several systematic reviews provide further evidence of the preventive effect of ‘11+’ especially in youth amateur football [10–13]. However, so far, no study has investigated the prevention of football injuries in children under the age of 14 years [14]. Compliance has been discussed as an important factor. On the one hand, the actual compliance is key to correctly interpreting the preventive effect of an intervention in the study setting. On the other hand, a high compliance is crucial to reach as many people as possible. The highest possible efficacy of the intervention combined with the highest possible compliance leads to the best possible injury reduction in the study and real-life setting [15–17].

Based on the ‘11+’ programme and age-specific epidemiological data [4, 5], an international group of experts developed and pilot tested an injury prevention programme for 7- to 13-year-old children (‘11+ Kids’) [18]. The present study evaluated the efficacy of the ‘11+ Kids’ programme to reduce the incidence of injuries in 7- to 13-year-old football players. We hypothesised that the overall injury incidence would be reduced by at least one-third in the intervention group compared with a control group [14].

## Methods

### Study Design and Definitions

The study was designed as a two-armed, cluster-randomised controlled trial (level of evidence 1) according to the CONSORT statement guidelines [19, 20], and conducted as a multi-centre study in four countries (Switzerland, Germany, the Czech Republic and the Netherlands). The trial was registered in the ClinicalTrials.gov registry (NCT02222025). Clubs who agreed to participate in the study were randomised to an intervention group (INT) or a control group (CON), and followed for one season.

Clubs of the INT were instructed to use the new injury prevention programme (‘11+ Kids’) as a warm-up, while CON clubs should warm up as usual. The CON clubs were informed that they would receive the programme after the end of the study if it proved efficacious in preventing injuries.

Injury characteristics and football exposure were assessed using guidelines for football injury research [21]. This refers to injury severity, location, type and diagnosis as well as definitions for training and match exposure. According to other studies on sport injuries in children and adolescents, the injury definition used was slightly adapted [22, 23]. An injury was defined as any physical complaint sustained by a child during a scheduled training session or match play resulting in (a) the inability to complete the current match or training session and/or (b) the absence from subsequent training sessions or matches and/or (c) the injury requiring medical attention [4].

## Observation Period

The observation period comprised one football season from August/September 2014 to June/July 2015.

### Study Population and Recruitment

**Fig. 1:**
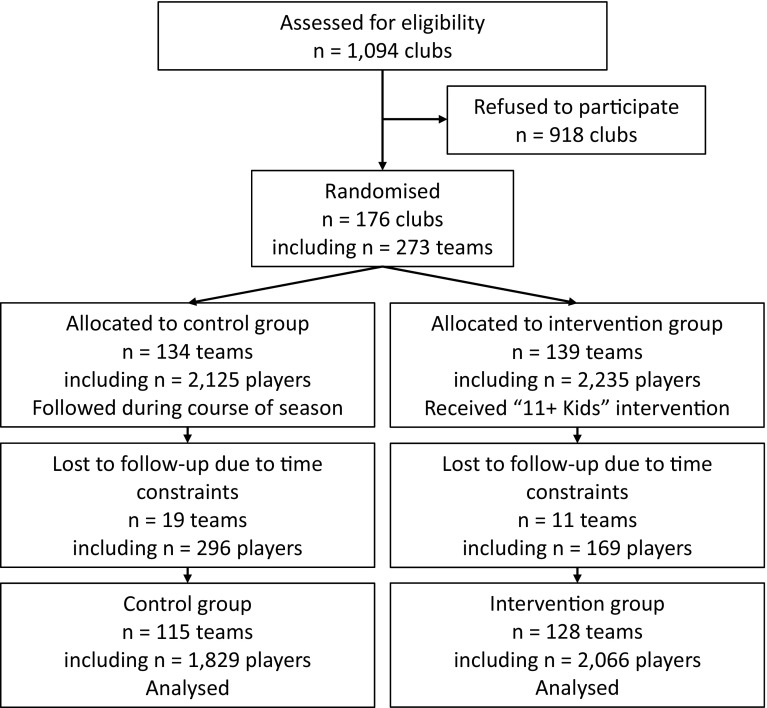
Flow of study participants

Between May and July 2014, 1094 officially registered clubs with teams in the age categories under 9 years, under 11 years and under 13 years (boys and girls, born 2002–7) were invited to participate in the study in Switzerland, Germany, the Czech Republic and the Netherlands (Fig. 1). Inclusion criteria were: (1) the club must be officially registered in the (regional) football association; (2) children must be between 7 and 12 years of age at the start of the study; and (3) regular training must take place at least twice per week. Teams were not eligible for inclusion if the coach already used an injury prevention programme or a structured warm-up focusing on neuromuscular control. Prior to the start of the study, information meetings were conducted to inform coaches about the aims and procedures of the study and, for INT teams only, to give detailed instructions and practical application on the ‘11+ Kids’ programme.

### Ethics

The study complied with ethical standards and the Declaration of Helsinki and was approved by the lead ethics committee (Ethikkommission Nordwest- und Zentralschweiz, EKNZ, Approval number 2014–232) and by all other regional ethics committees (Saarbrücken, Prague and Amsterdam). All children and their parents received written information about the aim and the methodology of the project prior to the start of the study. Participation was voluntary. Passive informed consent was acquired to include children into the statistical analysis. In the case where children or parents declined participation, parents informed the researchers via e-mail or telephone. All parents of injured children gave their active consent.

### Randomisation

Participating clubs were randomised into INT or CON. All teams of the same club were randomised into the same group (clustered allocation with the club serving as a cluster) to minimise the risk of contamination. Computer-generated cluster randomisation was conducted by one researcher (OF) who had no direct contact with the clubs or team officials and who was not involved in the intervention. Age group, country and number of participating teams per club served as the strata for the randomisation.

### Intervention

‘11+ Kids’ is an exercise-based programme to prevent football injuries in 7- to 13-year-old children. It was developed by an international group of experts based on the findings of an epidemiological study on injury incidence and characteristics in children’s football [4, 5]. The structure of the programme refers to the established ‘11+’ programme, which has been shown to be efficacious in players aged older than 13 years [7–9, 16].

A study on the preliminary version of this programme showed slight improvements in motor performance [18]. Such improvements have been described as a prerequisite for successful injury reduction [16, 24]. In addition, the programme proved feasible, and its acceptance was high among coaches and players (unpublished data). The results of this pilot study provided input for final programme adaptations. Prior to the start of the study, two teams (that did not take part in the study) extensively pilot tested the final programme.

‘11+ Kids’ consists of seven different exercises and can be performed in about 15–20 min after familiarisation. Three exercises focus on unilateral, dynamic stability of the lower extremities (hopping, jumping and landing), three exercises on whole body and trunk strength/stability, and one exercise on falling technique. The difficulty of each exercise is progressively increased in five levels to account for the varying age- and maturity-related performance levels, as well as for general differences in motor skills of children aged 7–13 years [see ‘11+ Kids’ manual, Electronic Supplementary Material (ESM) 1 and 2]. Coaches were instructed to start with the first level of each exercise and to proceed with the next level when all players were able to perform the exercise according to the description in the manual. Hereby, specific attention was set on the body alignment during the exercises (e.g. leg alignment during single-leg jumps).

During the first weeks of the season, our study assistants visited INT clubs and gave the coaches an instruction session on how to apply the ‘11+ Kids’ programme correctly. Coaches received a detailed manual of the ‘11+ Kids’ and a two-page summary for the pitch, and were advised to use the ‘11+ Kids’ programme at the beginning of their training sessions as a replacement of their usual warm-up at least twice a week. The coaches of CON were instructed to perform their usual warm-up.

### Injury Surveillance and Documentation of Football Exposure

Player-specific football exposure (in minutes), sustained injuries and session-based information about ‘11+ Kids’ utilisation (INT only) were collected using an Internet-based injury registration system. This online platform was developed for (and successfully applied in) a previous epidemiological study in children’s football [4, 5]. It had been adapted based on previous experiences to improve the usability of the system and, thereby, to increase the compliance of the coaches. Self-reported anthropometric baseline data of the children were provided by parents at the start of the study. One contact person for each team (preferably, but not necessarily the coach) was appointed and instructed to complete the injury and exposure entry into the online injury recording system. The documentation of exposure time and injuries in INT teams started after the instruction session.

In case no data were entered within a period of 1 week, an automated reminder e-mail was sent. If an injury occurred, trained study assistants contacted the coach, the player and the parents via telephone and/or e-mail to assess all relevant aspects of the injury based on a standardised injury registration form. If an injury received medical treatment, parents were instructed to obtain the exact diagnosis from the treating physician. All information on each injury was screened by two medically trained investigators (MB, KadF), who were blinded to group allocation, to ensure an objective and independent injury classification.

To ensure good compliance regarding entry of exposure and injury data, four scientific assistants (one in each country) and nine study assistants supported coaches during data collection and injury recording. Each study assistant was responsible for 10–15 clubs. Study assistants were continuously in touch with the coaches via telephone and e-mail, and visited unannounced two training sessions of each team during the study period.

### Evaluation of Feasibility and Acceptance Among Coaches

Study participants (coaches and players) were involved in the design of the intervention programme. In a previous (pilot) study [18], the intervention programme has been evaluated by coaches and players. Their valuable feedback has been considered during the development of the final version of the intervention programme, which was used in the study at hand.

Furthermore, in this study, the intervention programme has been evaluated by the coaches. The INT coaches were asked to complete an online questionnaire about their general rating of the programme (13 questions on e.g. quality of the manual, time requirement) and each of the seven exercises (five questions per exercise) at the end of the study (see ESM 3). A five-level Likert scale was used as described earlier [25].

### Sample Size

For the primary outcome (overall injuries), sample size estimation revealed that a total of 1935 children are needed to detect a hypothetical hazard ratio (HR) of 0.66 with a power of 80%, and an alpha level of 0.05. This is based on the assumption that the club (cluster) contains 40 players on average, the intraclass correlation coefficient is 0.05, that 7% of the players in the control group will sustain an injury during the season and taking into account a design effect (inflation factor of 2.95) [4, 9, 26, 27].

### Statistical Procedure

Player-specific time-to-injury data were analysed using extended Cox models. Uninjured children contributed their right censored ‘survival times’ to the analysis. The models contained mixed (random and fixed) effects. To acknowledge the clustered data structure, we included the variable ‘club’ and ‘team’ as a random effect. Further, to acknowledge that multiple injuries of one player are not independent, we also included the variable ‘id’ (player-specific identifier) as a random effect. The models were fit to reflect that “teams are nested in clubs”, “players are nested in teams” and “multiple injuries of one player are nested in the player”. This approach has been used previously [5]. During model building, we also fitted models to include the ‘country level’ to account for the hierarchical structure (i.e. “clubs located in countries”). To decide whether to include the additional level, we compared the integrated log-likelihood value with the less complex model (i.e. containing ‘team’ and ‘id’ level only). The Chi square tests showed large *p* values (*p* ≥ 0.58) for the comparisons between models. We therefore decided to use these less complex models (including ‘team’ and ‘id’ level) [28–30].

The proportional hazard assumption was tested during model building [31]. The intervention variable (INT vs. CON) was used as a fixed effect. Further, we entered variables (age, age-independent body height, age-independent body mass and match-training ratio) that had *p* < 0.2 in the univariate analysis into the multivariate model [5, 32]. When multicollinearity between two variables was present, we included the one with the smaller *p* value into the multivariate analysis.

The analyses were performed using R (Version 3.2.2) in combination with RStudio (Version 0.99.484) in a cloud computing environment on multiple servers. We used the ‘coxme’ package (Version 2.2-5) to fit the models. Kaplan–Meier curves were plotted for overall, severe and lower extremity injuries.

To conduct a compliance analysis, we carried out a tertile split of the INT players according to their weekly ‘11+ Kids’ completion rate [8]. “Completion” was defined as the full utilisation of the ‘11+ Kids’ warm-up programme (with all of its seven exercises as described in the manual) at the beginning of a training session. We compared the three INT groups (high/middle/low compliance) against each other as well as against CON using extended Cox models. We used player-specific ‘11+ Kids’ completion data (rather than team-based information). Therefore, the actual individual exposure to the intervention programme was taken into account.

In addition, we used the aforementioned extended Cox models in a different approach to investigate the influence of compliance (completed ‘11+ Kids’ training sessions per week) on the reduction of injury rate. Thereby, a compliance threshold was increased stepwise by 0.01 increments, removing all players with a compliance below this threshold. Our intention was to evaluate the benefit from additional weekly sessions.

Finally, we applied the magnitude-based inference approach to investigate the intervention effect regarding specific types, locations and mechanisms of injuries. It has to be mentioned that the study was not powered for these subgroup analyses. However, it might provide useful information about the clinical relevance of the effects found. We used an open source spreadsheet to run the analyses [33]. Hazard ratios and the associated *p* values were used to get 90% confidence limits for, and inferences about, the true value of an effect statistic. Threshold values for “benefit” HR < 0.77 and “harm” > HR 1.30 were used. Qualitative descriptors were assigned to quantitative chances of intervention effects as follows: 0.5–5%: “very unlikely”; > 5–25%: “unlikely”; > 25–75%: “possibly”; > 75–95% “likely”; > 95–99.5%: “very likely”; > 99.5%: “almost certainly” [34].

## Results

### Main Analysis

In total, 292,749 h of football exposure [7026 h (2.4%) completed by girls] of 3895 players [*n* = 171 (4.4%) girls] were recorded. The mean age of players was 10.8 (standard deviation 1.4) years. The INT and CON players were of similar age, body mass and height. Further baseline data are presented in Table 1.

**Table 1:** Player and injury characteristics of the control (CON) and intervention (INT) groups

	CON	INT
Number of teams	115	128
Number of players	1829	2066
Age [years]	10.7 (1.4)	10.8 (1.4)
Body height [m]	1.44 (0.10)	1.45 (0.11)
Body mass [kg]	36.4 (8.5)	36.3 (8.5)
BMI [kg/m^2^]	17.3 (2.5)	17.1 (2.4)
Total exposure [h]	152,033	140,716
Match exposure [h]	23,813	19,769
Training exposure [h]	128,220	120,947
Number of total injuries	235	139
Number of match injuries	115	71
Number of training injuries	120	68
Total number of injured players	184	119
Number of players with 1 injury	149	102
Number of players with 2 injuries	22	14
Number of players with 3 injuries	10	3
Number of players with 4 injuries	3	0
Number of “recurrent” injuries^a^	15	6
Number of injuries by time loss (%)		
No time loss	18 (7.7)	8 (5.8)
1–3 days	37 (15.7)	23 (16.5)
4–7 days	52 (22.1)	41 (29.5)
8–28 days	78 (33.2)	46 (33.1)
>28 days	50 (21.3)	21 (15.1)
Sum of days lost to injury Mean lay-off time with 95% CI [days]	4201 17.9 [15.0–20.8]	2026 14.6 [11.6–17.6]

*BMI* body mass index, *CI* confidence interval

^a^Re-injury of the same body part (e.g. “a second sprain of the left ankle”)

**Fig. 2:**
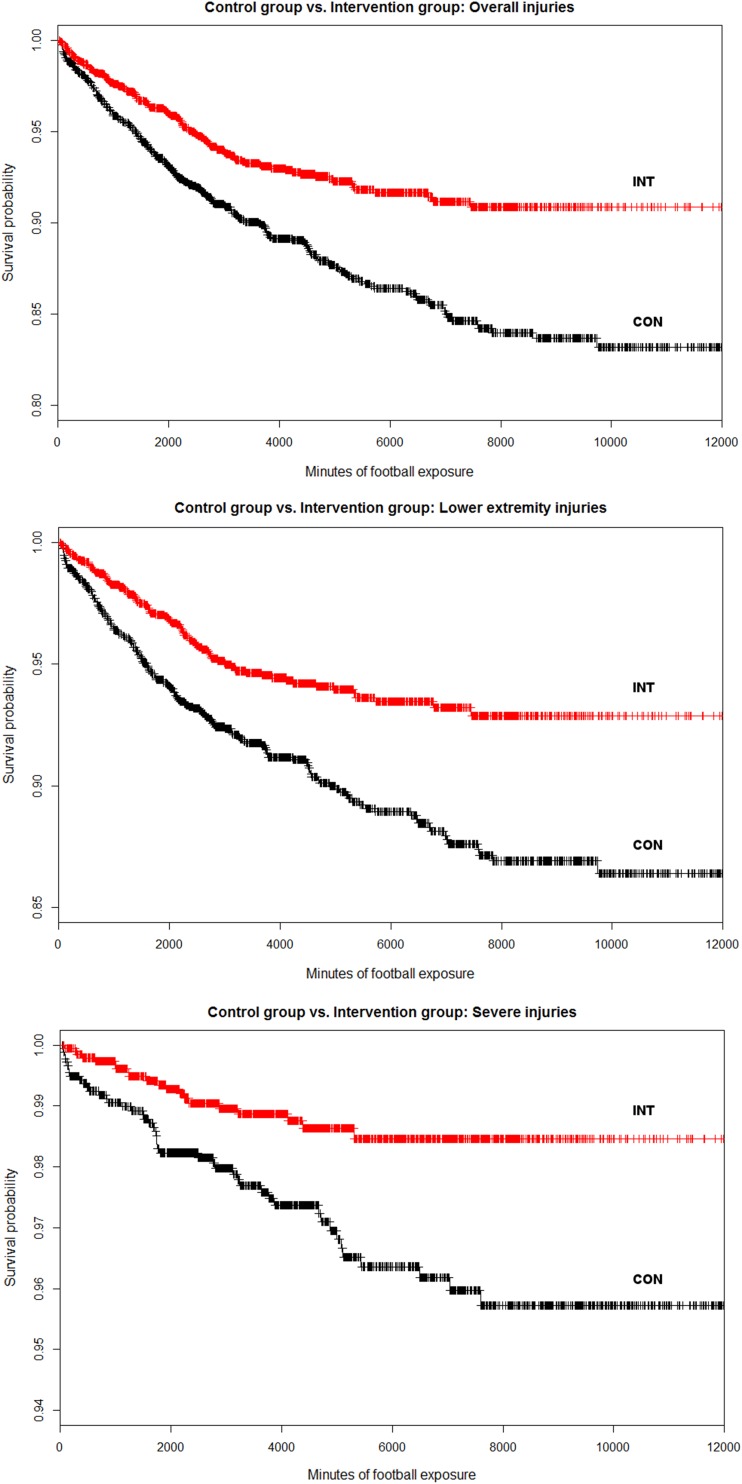
Kaplan–Meier plots of the control group (CON; performing their regular warm-up) and the intervention group (INT; performing ‘11+ Kids’ as a warm-up). The plot on the top shows the ‘survival probability’ regarding overall injuries, the middle plot shows the lower extremity injuries and the lower plot shows the severe injuries (resulting in > 28 days of lay-off time)

The overall injury rate in INT was reduced by 48% compared with CON [HR 0.52; 95% confidence interval (CI) 0.32–0.86]. Severe injuries (HR 0.26; 95% CI 0.10–0.64) and lower extremity injuries (HR 0.45; 95% CI 0.24–0.84) were also reduced (Fig. 2). Additional reductions were found regarding match injuries (HR 0.51; 95% CI 0.27–0.94) and training injuries (HR 0.58; 95% CI 0.38–0.89). Mean lay-off time and the total number of days lost because of injury were lower in INT (Table 1).

The number and incidence of injuries in INT and CON by location, type and injury mechanism are presented in Table 2. Knee, ankle, thigh and hip/groin injuries were less frequent in INT with HRs ranging between 0.40 and 0.52. Joint/ligament, muscle injuries, traumatic fractures, as well as overuse-related complaints showed HRs between 0.12 and 0.56. Additionally, running/jumping, overuse/growth and collision-related injuries were lower in INT with HRs between 0.30 and 0.52 (Table 2).

**Table 2:** Number of injuries (*N*), percent of total injuries (%), injury incidence rate (IR; per 1000 h of exposure) in control (CON) and intervention (INT) groups, results of the mixed-effects Cox-regression analyses (hazard ratio, HR; adjusted for team and intra-person clustering, age, age-independent body height and match-training ratio), and outcomes of the magnitude-based inference approach (based on 90% confidence limits and threshold values for “benefit” HR < 0.77 and “harm” > HR 1.30). Qualitative descriptors: 0.5–5%: “very unlikely”; > 5–25%: “unlikely”; > 25–75%: “possibly”; > 75–95% “likely”; > 95–99.5%: “very likely”; > 99.5%: “almost certainly”. The table shows the intervention effects regarding specific locations, types and mechanisms of injuries

	CON (*n* = 235 injuries)			INT (*n* = 139 injuries)			HR [95% CI]	Inference	% Likelihood effect is beneficial | trivial | harmful
	*N*	%	IR	*N*	%	IR			
Location									
Knee	54	23.0	0.355	29	20.9	0.206	0.47 [0.19–1.13]	Likely beneficial, very unlikely harmful	86.7 | 12.2 | 1.1
Ankle	44	18.7	0.289	26	18.7	0.185	0.52 [0.22–1.22]	Likely beneficial, very unlikely harmful	81.8 | 16.5 | 1.7
Finger/hand/wrist/arm	28	11.9	0.184	24	17.3	0.171	0.91 [0.47–1.76]	Unclear	31.0 | 54.4 | 14.6
Thigh	29	12.3	0.191	17	12.2	0.121	0.44 [0.14–1.35]	Likely beneficial, very unlikely harmful	83.7 | 13.4 | 2.9
Foot	23	9.8	0.151	18	12.9	0.128	0.69 [0.31–1.53]	Unclear	60.7 | 33.4 | 5.9
Lower leg/achilles tendon	20	8.5	0.132	10	7.2	0.071	0.58 [0.21–1.62]	Unclear	70.5 | 23.2 | 6.3
Hip/groin	16	6.8	0.105	4	2.9	0.028	0.40 [0.12–1.34]	Likely beneficial, very unlikely harmful	85.4 | 11.7 | 2.9
Head/face/neck	8	3.4	0.053	5	3.6	0.036	0.71 [0.09–5.59]	Unclear	53.0 | 18.3 | 28.7
Shoulder/clavicle	4	1.7	0.026	2	1.4	0.014	0.21 [0.01–13.15]	Unclear	73.1 | 7.5 | 19.4
Lower back	4	1.7	0.026	1	0.7	0.007	0.50 [0.01–26.16]	Unclear	58.5 | 9.8 | 31.7
Upper trunk	3	1.3	0.020	2	1.4	0.014	1.07 [0.15–7.71]	Unclear	35.7 | 22.8 | 41.5
Buttock	2	0.9	0.013	1	0.7	0.007	0.47 [0.04–5.58]	Unclear	65.2 | 13.7 | 21.1
Type									
Contusion	54	23.0	0.355	45	32.4	0.320	0.66 [0.33–1.34]	Unclear	66.5 | 30.4 | 3.0
Joint/ligament injury	47	20.0	0.309	31	22.3	0.220	0.56 [0.24–1.28]	Likely beneficial, very unlikely harmful	77.4 | 20.2 | 2.3
Muscle injury	42	17.9	0.276	18	12.9	0.128	0.46 [0.17–1.21]	Likely beneficial, very unlikely harmful	85.5 | 12.8 | 1.6
Fracture traumatic	32	13.6	0.210	16	11.5	0.114	0.55 [0.27–1.13]	Likely beneficial, very unlikely harmful	82.3 | 16.8 | 0.9
Overuse	24	10.2	0.158	4	2.9	0.028	0.12 [0.01–1.13]	Likely beneficial, very unlikely harmful	94.8 | 3.3 | 1.8
Other	13	5.5	0.085	12	8.6	0.085	0.76 [0.21–2.69]	Unclear	50.8 | 28.9 | 20.3
Growth-related complaints	14	6.0	0.092	7	5.0	0.050	0.81 [0.19–3.53]	Unclear	47.3 | 26.1 | 26.6
Abrasion	6	2.6	0.039	4	2.9	0.028	1.48 [0.25–8.86]	Possibly harmful, unlikely beneficial	23.9 | 20.5 | 55.6
Concussion	2	0.9	0.013	1	0.7	0.007	0.77 [0.04–13.61]	Unclear	50.0 | 13.8 | 36.2
Dental	1	0.4	0.007	1	0.7	0.007	0.96 [0.06–15.98]	Unclear	44.6 | 12.7 | 42.6
Mechanism									
Duel	42	17.9	0.276	22	15.8	0.156	0.73 [0.26–2.05]	Unclear	54.0 | 32.3 | 13.7
Foul	29	12.3	0.191	25	18.0	0.178	0.68 [0.28–1.65]	Unclear	60.7 | 31.4 | 7.9
Running/jumping	37	15.7	0.243	16	11.5	0.114	0.38 [0.13–1.16]	Likely beneficial, very unlikely harmful	89.2 | 9.2 | 1.6
Overuse/growth	38	16.2	0.250	11	7.9	0.078	0.52 [0.18–1.34]	Likely beneficial, very unlikely harmful	81.2 | 16.8 | 1.9
Contact with object	14	6.0	0.092	15	10.8	0.107	1.00 [0.40–2.51]	n.a.	n.a.
Collision	20	8.5	0.132	7	5.0	0.050	0.30 [0.09–0.98]	Likely beneficial, very unlikely harmful	94.1 | 5.2 | 0.8
Other	14	6.0	0.092	13	9.4	0.092	0.86 [0.27–2.79]	Unclear	40.4 | 41.4 | 18.2
Cutting	18	7.7	0.118	8	5.8	0.057	0.61 [0.22–1.68]	Unclear	67.7 | 25.5 | 6.8
Falling	14	6.0	0.092	10	7.2	0.071	0.82 [0.30–2.27]	Unclear	45.1 | 36.3 | 18.6
Header duel	8	3.4	0.053	9	6.5	0.064	0.44 [0.10–1.89]	Unclear	77.4 | 15.3 | 7.3
Kicking the ball	1	0.4	0.007	3	2.2	0.021	2.95 [0.30–28.69]	Likely harmful, unlikely beneficial	12.3 | 11.6 | 76.1

*CI* confidence interval, *n.a. *not applicable

### Compliance Analysis

Injury incidence decreased with the increasing utilisation rate of the ‘11+ Kids’ programme. The risk of injury was lower in the high-compliance group and the middle-compliance group compared with CON. The risk of injury in the high-compliance group was half compared with the low-compliance group (Table 3).

**Table 3:** Results of the mixed-effects Cox-regression analyses (adjusted for team and intra-person clustering, age, age-independent body height and match-training ratio) comparing different compliance groups [tertile split according to ‘11+ Kids’ sessions per week: low, middle, high compliance (LOW, MID, HIGH)] and the control group (CON)

	CON	LOW	MID	HIGH
IR per 1000 h [95% CI]	1.56 [1.38–1.78]	1.25 [0.80–1.95]	0.95 [0.64–1.42]	0.62 [0.42–0.91]
Sessions per week mean (SD)		0.6 (0.1)	0.9 (0.1)	1.5 (0.4)
Sessions per week range		0.3–0.8	0.8–1.1	1.1–2.9
Comparisons HR [95% CI]				
CON	1			
LOW	0.68 [0.40–1.15]	1		
MID	0.62 [0.40–0.97]	0.64 [0.39–1.06]	1	
HIGH	0.44 [0.28–0.69]	0.50 [0.29–0.84]	0.77 [0.46–1.30]	1

*CI* confidence interval, *HR* Hazard ratio, *IR* injury rate, *SD* standard deviation

The extended Cox model revealed an influence of compliance on injury incidence rates (HR 0.77; 95% CI 0.64–0.92) within the sample of INT players. The effect remains consistent (HR 0.75; 95% CI 0.59–0.96) when adjusting for the known injury risk factors ‘age’, ‘age-independent body height’ and ‘and match-training ratio’ (i.e. the hours of match play divided by the number of training hours) of the players [5].

The compliance-threshold analysis revealed that the added benefit of each additional session per week was stable in players who performed the programme up to 0.75 times per week. Above this value, the influence of compliance increased and reached its highest additional benefit (per additional session) at 1.25 sessions per week.

### Coaches’ Evaluation

The descriptive statistics of the evaluation of ‘11+ Kids’ imply that coaches feel that injury prevention in general is important (86% of coaches fully agreed and gave 5 out of 5 points, 9% gave 4 points, and 5% gave 3 points). The quality of the ‘11+ Kids’ manual was rated high (83% gave 5, 10% gave 4, 3% gave 3, 2% gave 2, and 2% gave 1 point). Coaches believe that the programme can prevent injuries (29% gave 5, 34% gave 4, 34% gave 3, 2% gave 2, and 2% gave 1 point) and improve a player’s performance (10% gave 5, 38% gave 4, 41% gave 3, 10% gave 2, and 2% gave 1 point). The reported time requirement was 18.1 (standard deviation 4.4) min to perform the whole programme. Time requirement was rated as being just reasonable (21% gave 5, 30% gave 4, 19% gave 3, 24% gave 2, and 6% gave 1 out of 5 points) [see evaluation, ESM 3].

## Discussion

### Principal Findings

Using ‘11+ Kids’ as a warm-up reduced football injuries in 7- to 13-year-old children by 48% compared with the control group. Particularly large protective benefits were found for severe injuries (74%). The mean injury lay-off time was reduced in INT. Importantly, the total number of days lost to injury (i.e. absence from sport participation) was also considerably lower (less than half).

Regular and frequent use of the programme appears to be crucial to profit from the preventive effect and/or to increase this effect. The compliance analysis showed a clear dose–response relationship between the frequency of performing ‘11+ Kids’ and the injury rate. The programme should be used at least once every week to profit from a protective effect, and more often, to maximise the benefits of the intervention programme.

### Strengths and Weaknesses of the Study

To the best of our knowledge, this is the first study to investigate the effects of an injury prevention programme for organised football in children younger than 13 years of age. A general issue in exercise intervention studies is that blinding of participants regarding group allocation is nearly impossible. The percentage of girls in this study was representative for the involved national football associations. However, the low proportion of girls (and number of injuries sustained by girls) limits the transferability of the observed effects to the population of football-playing girls. However, comparable injury-preventive effects of the ‘11+’ have been described in young female and male players [8, 9]. Therefore, it might be speculated that the results found in the study at hand could be transferred to the population of football-playing girls.

We used extended Cox models to take into account individual hazards (frailties) and potential team-clustering effects. Although ‘frailty models’ have been used in medical applications (e.g. cancer research) for over 20 years [35], this approach is not established yet in the field of sport science and medicine. This procedure enabled us to analyse some subgroups of injuries (e.g. lower extremity injuries and severe injuries) and to investigate compliance with sufficient power.

Similar to comparable high-quality studies [6, 8, 9], data were reported by coaches and partly by parents and players. We aimed to improve the quality of reporting by the following means: at the beginning of the study, all coaches of INT and CON were trained and sensitised to injury definitions and regularly contacted by our study assistants to ensure timely and complete data entry. Throughout the whole season, they were contacted on a regular basis to improve compliance and completeness of documentation. To minimise a potential recall bias, coaches received an automated reminder e-mail within a week if they did not enter data into the online system. After 2 weeks without data entry, our study assistants contacted the coaches personally (via telephone and/or e-mail). Study assistants visited two training sessions of each intervention team (without previous announcement) to check whether they used the intervention programme.

For all injuries, parents and injured children were contacted to double check the information provided by the coaches. Only for about half of the injuries (those that were medically treated) medical diagnoses were available. Two blinded medical professionals checked the plausibility and consistency of injury data.

We used a very broad recruitment strategy in Switzerland (we contacted all 846 clubs in the German-speaking part of Switzerland per email) but not in the other countries. We did not observe relevant differences in injury rates and intervention effects between countries. Further, there were no differences regarding compliance to the intervention programme. Therefore, we are quite confident that the high level of refusal in Switzerland did not affect the outcomes of the study.

The exposure time was lower in INT than in CON because the documentation in the INT teams started after the instruction session. However, the proportional hazard assumption was fulfilled in both groups. Further, we performed a sensitivity analysis by cutting the respective time period (exposure time and injury events) in the control group at the beginning of the season. The results were similar compared to the regular analysis. Consequently, the estimate of the intervention effect was very likely not biased.

Dropout rate was higher in CON (13.9%) than in INT (7.6%). This might be owing to the fact that CON coaches only had “additional work” (i.e. data entry) without having a “benefit” (i.e. receiving the intervention programme). In turn, the lower dropout rate in INT might be interpreted in favour of the intervention programme (structure and content as well as feasibility).

In addition to a standard compliance analysis (i.e. comparison of compliance groups based on tertile split) [8], we introduced a novel and exploratory approach to further investigate the influence of compliance. To the best of our knowledge, this is the first study applying this statistical approach. Compliance was assessed with the following question: “Did you perform the ‘11+ Kids’ programme as suggested in the manual?” (answer: yes/no). We have chosen this ‘simple’ approach because of feasibility for the coaches. Based on the experience with our previous large-scale epidemiological study [4, 5], we knew that it would be critical to keep coaches’ time expenses as low as possible to avoid additional dropout. Detailed questions about programme utilisation (e.g. asking whether each of the seven exercises have been performed with the correct number of repetitions and with the correct technique) might have been too complex and time consuming for many coaches.

We observed a wide spectrum of programme utilisation (i.e. ranging from 0.3 to 2.9 sessions per week). These data do not indicate an artificially high compliance (i.e. due to social desirability bias) and therefore appear to be plausible. Hence, we are confident that coaches reported honestly whether they actually used the intervention programme or not.

### Comparison to Other Studies

The overall reduction of injuries in the present study is similar to studies in older football players, and in other youth team sports [14]. From a ‘public health’ perspective, it has been argued that injury prevention should focus on different aspects: the most common, the most severe, the most debilitating or the most costly injuries [14, 36]. The reduction in severe injuries was even higher compared with studies in older athletes [14]. The reduction in lower extremity injuries (55%) was similar to other studies [14, 37].

Remarkable injury reductions have been observed in several studies focusing on specific types of injuries [14]. Furthermore, in the study at hand, some specific subgroups of injuries regarding location (knee, ankle, thigh and hip/groin injuries), type (joint/ligament, muscle injuries, traumatic fractures and overuse-related complaints) and mechanism (running/jumping, overuse/growth and collision-related injuries) showed low HRs. The corresponding 95% CIs for the HRs of the latter subgroups are mostly too wide to draw firm conclusions. The magnitude-based inferences, however, indicate ‘likely beneficial’ intervention effects.

Compliance has shown to be important regarding exercise-based injury prevention, as beneficial effects were greater in players with higher compliance to the programme [16, 38, 39]. We also observed that injury incidence was reduced with increased compliance. The effects are comparable to those observed in female youth football players (13–17 years of age) [38].

The survey among INT coaches revealed that the quality of the ‘11+ Kids’ manual and the feasibility of the programme were rated high. The time requirement has been rated as being just reasonable. Time constraints and a perceived inappropriateness of the exercises have been discussed as potential barriers that might lead to low compliance [38].

### Practical Relevance of the Study

Playing football can induce considerable health benefits, and thus, football has a great potential to support a healthy lifestyle from a young age onwards [40, 41]. Injuries have been reported to be one of the most relevant reasons to drop out from sport participation [42]. Successful injury prevention can reduce the number of dropouts, apart from providing the obvious (and direct) health benefits of staying injury free. Thus, injury prevention can support children achieving higher physical activity levels with all its positive health effects as it allows for more consistent sport participation [43, 44]. Negative health consequences are not limited to the short term. The early development of osteoarthritis is one example of a harmful long-term effect [45, 46]. It can reasonably be assumed that early injury prevention may support long-term health benefits. Therefore, it is recommended to implement injury prevention in football starting at a young age.

### Future Research

Future studies might investigate the effectiveness of the programme in a large-scale day-to-day application. Further, the transferability of ‘11+ Kids’ to other (team) sports should be explored [47]. Last, but not least, the cost effectiveness (cost of application in relation to the reduction of healthcare costs) should be analysed in subsequent studies.

## Conclusion

The new warm-up programme ‘11+ Kids’ has proven efficacious in reducing injury rates in children’s football by almost 50%. The more frequently players performed ‘11+ Kids’ (i.e. higher compliance), the greater was the observed injury reduction. The warm-up programme should be used at least once per week; however, two sessions per week are recommended to further increase the protective benefit. Coaches rated the quality of the ‘11+ Kids’ manual and the feasibility of the programme high, and the time requirement as being just reasonable. ‘11+ Kids’ should be implemented on a large scale to reduce injuries and their potential negative effects on sport participation and long-term health.

## Electronic supplementary material

Below is the link to the electronic supplementary material.

‘11+ Kids’ manual (study version)

‘11+ Kids’ short version of the manual (study version)

Table 1: General evaluation of ‘11+ Kids’ by coaches of the intervention group at the end of the season. For items 1–11, a five-level Likert scale was used as follows: 1: Strongly disagree, 2: Disagree, 3: Neither agree nor disagree, 4: Agree, 5: Strongly agree. Table 2: Exercise-specific evaluation of ‘11+ Kids’ by coaches of the intervention group at the end of the season. For items 1–3, a five-level Likert scale was used as follows: 1: Strongly disagree, 2: Disagree, 3: Neither agree nor disagree, 4: Agree, 5: Strongly agree
