# Supplementary material for: A Multinational Cluster Randomised Controlled Trial to Assess the Efficacy of ‘11+ Kids’: A Warm-Up Programme to Prevent Injuries in Children’s Football
Source: Sports Med. 2017 Dec 22;48(6):1493–504. doi: 10.1007/s40279-017-0834-8 (PMC5948238; doi:10.1007/s40279-017-0834-8)
Supplement: Supplementary file 1 — ‘11+ Kids’ manual (study version) [file 40279_2017_834_MOESM1_ESM.pdf]

Sports Medicine ISSN: 0112-1642 (Print) 1179-2035 (Online) Springer International Publishing

A Multinational Cluster Randomised Controlled Trial to Assess the Efficacy of '11+ Kids': A Warm-Up Programme to Prevent Injuries in Children's Football

Short title: Injury prevention in children's football

Roland Rössler[1,2*] (RR, postdoc), Astrid Junge[3,4,5] (AJ, professor), Mario Bizzini[3,4] (MB, physiotherapist), Evert Verhagen[2] (EV, assistant professor), Jiri Chomiak[6] (JC, medical doctor), Karen aus der Fünten[7] (KadF, medical doctor) Tim Meyer[7], (TM, professor), Jiri Dvorak[3,4] (JD, professor), Eric Lichtenstein[1] (EL, research assistant), Florian Beaudouin[7] (FB, research assistant), Oliver Faude[1] (OF, senior researcher)

1 Department of Sport, Exercise and Health University of Basel, Basel Switzerland

2 Amsterdam Collaboration on Health & Safety in Sports and Department of Public and Occupational Health, Amsterdam Movement Science VU University Medical Center, Amsterdam Netherlands

3 Swiss Concussion Center, Zurich Switzerland

4 Schulthess Clinic, Zurich Switzerland

5 Medical School Hamburg, Hamburg Germany

6 Orthopaedic Department 1st Faculty of Medicine Charles University and IPVZ and Hospital Na Bulovce and FIFA med. Centre, Prague Czech Republic

7 Institute of Sports and Preventive Medicine Saarland University, Saarbrücken Germany

* Corresponding author

Roland Rössler, PhD

University of Basel

Department of Sport, Exercise and Health

Birsstrasse 320B

4052 Basel Switzerland

roland.roessler@unibas.ch

ORCID: 0000-0002-6763-0694

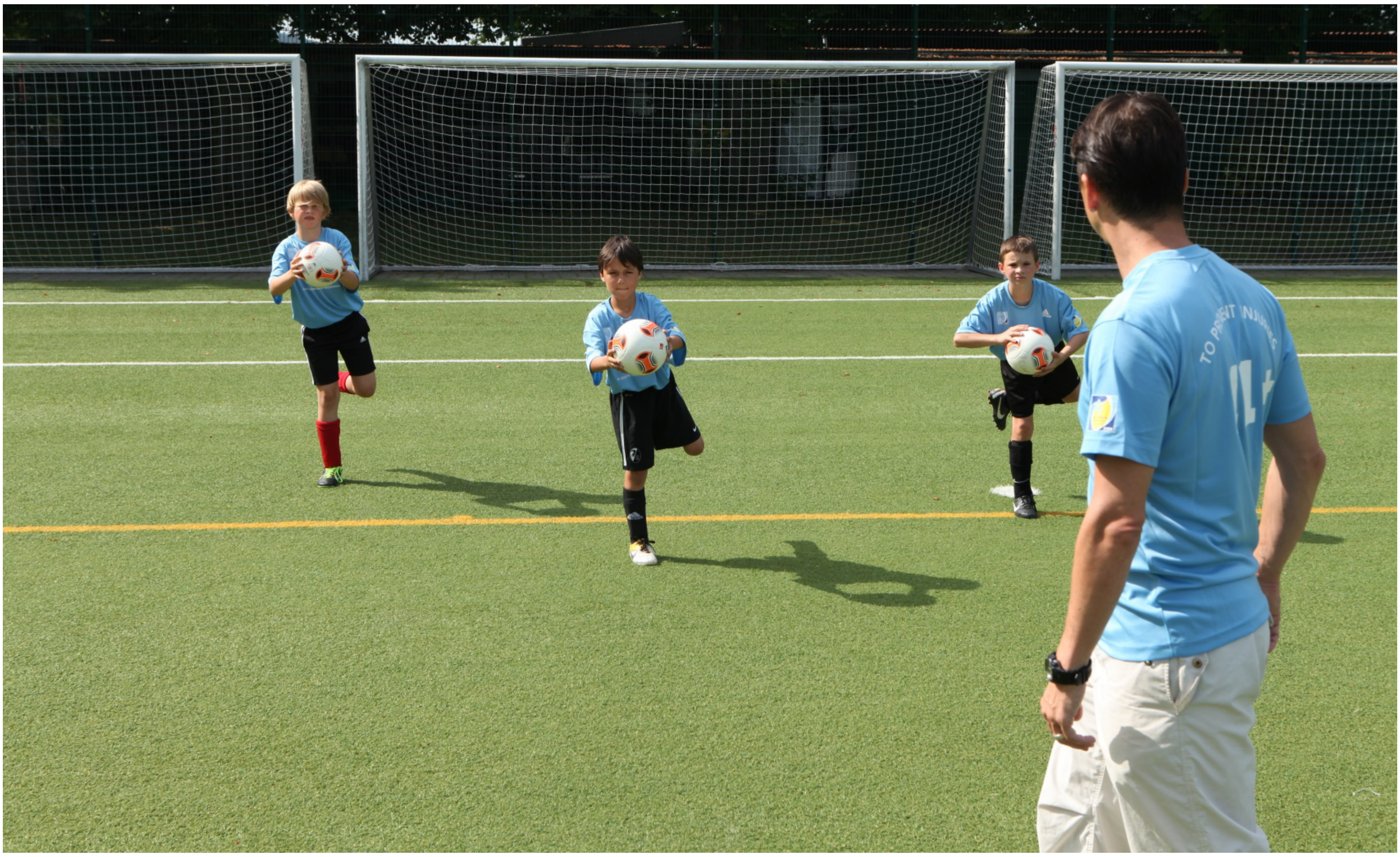

FIFA 11+ Kids

Ein Aufwärmprogramm

zur Verletzungsprävention im Kinderfussball

Manual für den Trainer

F-MARC
FOOTBALL FOR HEALTH
FIFA

© 2007 FIFA TM

Entwicklung und inhaltliche Konzeption

PD Dr. Oliver Faude (Departement für Sport, Bewegung und Gesundheit, Universität Basel, Schweiz)

M. Sc. Roland Rössler (Departement für Sport, Bewegung und Gesundheit, Universität Basel, Schweiz)

Dr. Mario Bizzini (FIFA Medical Assessment and Research Centre, Zurich, Switzerland)

Prof. Dr. Evert Verhagen (VU University Medical Center Amsterdam, The Netherlands)

Prof. Dr. Astrid Junge (FIFA Medical Assessment and Research Centre, Zurich, Switzerland)

Unter Mitarbeit von

Dr. Karen aus der Fünten (Institut für Sport- und Präventivmedizin, Universität des Saarlandes, Saarbrücken, Deutschland)

Prof. Dr. Jiri Chomiak (Department of Orthopaedics, 1st Faculty of Medicine, Charles University and Hospital, Prague, Czech Republic)

Prof. Dr. Tim Hewett (The Ohio State University Wexner Medical Center, Ohio, United States)

M. Sc. Nicolas Mathieu (Bereich Physiotherapie Fachhochschule Westschweiz, Leukerbad, Schweiz)

M. Sc. Ralf Roth (Departement für Sport, Bewegung und Gesundheit, Universität Basel, Schweiz)

F-MARC FOOTBALL FOR HEALTH FIFA

© 2007 FIFA TM

DSBG UNIVERSITY OF BASEL Department of Sport, Exercise and Health

Inhaltsverzeichnis

Inhaltsverzeichnis

Einleitung 4

Inhalt und Aufbau 4

Erreichen des nächsten Levels 5

Organisation auf dem Platz 5

Korrekte Ausführung und Kernelemente 6

Übung 1: Laufspiel „Wachmann“ 8

Übung 2: Skating-Sprünge 12

Übung 3: Einbeinstand 16

Übung 4: Liegestütz 20

Übung 5: Einbeinsprünge 24

Übung 6: Spiderman 28

Übung 7: Seitliches Abrollen 32

Der folgende Text meint Mädchen/Frauen und Jungen/Männer gleichermassen. Aufgrund der Lesbarkeit wird die männliche Form verwendet.

Einleitung

Beim Fussballspiel sind Ausdauer, Schnelligkeit, Beweglichkeit und Kraft sowie technisches und taktisches Spielverständnis leistungsbestimmend. Durch gezieltes Training werden diese Fertigkeiten verbessert. Fussballspielen hat viele positive Effekte auf die Gesundheit, doch es birgt auch ein gewisses Verletzungsrisiko. Deshalb sollte ein ganzheitliches Training Übungen zur Verletzungsprävention beinhalten. Das neue Verletzungspräventionsprogramm „FIFA 11+ Kids" wurde von einer internationalen Expertengruppe für Kinder im Alter von 7 bis 12 Jahren entwickelt. Es ist ein Aufwärmprogramm und ersetzt das herkömmliche Aufwärmen vor dem Training. Das Programm zielt auf eine Reduktion der Risikofaktoren ab, die zu Verletzungen führen können. Zudem kann FIFA 11+ Kids die motorische Leistungsfähigkeit der Spieler verbessern.

Inhalt und Aufbau

FIFA 11+ Kids fokussiert drei wichtige Bereiche der Verletzungsprävention:

- Verbesserung von Koordination und Gleichgewicht
- Kräftigung von Bein- und Rumpfmuskulatur
- Optimierung von Falltechniken

Das Programm besteht aus insgesamt sieben Übungen, die in der angegebenen Reihenfolge zu Beginn jedes Trainings durchgeführt werden sollen. Für jede Übung gibt es fünf aufeinander aufbauende Schwierigkeitsstufen (Level 1 bis 5). Beginnen Sie mit Level 1. Die Anweisungen für die Kinder sollen so kurz, klar und deutlich wie möglich sein. Achten Sie auf eine korrekte Körperhaltung und eine gute Körperkontrolle:

- Gerade Beinachse: Das Knie ist leicht gebeugt und weicht nicht seitlich aus. Die Fussspitze zeigt nach vorne.
- Körperspannung: Bauch- und Rückenmuskulatur sind angespannt. Der Rücken ist gerade und der Kopf in Verlängerung der Wirbelsäule.

Korrigieren Sie alle Fehler sorgfältig! Hinweise dazu sind bei den einzelnen Übungen beschrieben. Zu Beginn sollten die Wiederholungszahlen und Distanzen reduziert werden. Erst wenn die Übung korrekt ausgeführt wird, sollten die Dauer bzw. die Anzahl der Wiederholungen bis zur vorgeschlagenen Intensität erhöht werden.

Die Bilder zu den einzelnen Übungen verdeutlichen den Bewegungsablauf. Sie sind von links nach rechts in chronologischer Abfolge angeordnet. Das heisst die Startposition ist links und die Endposition rechts abgebildet.

Erreichen des nächsten Levels

Ein Spieler kann mit dem nächsten Level beginnen, wenn die Übung in mehreren aufeinanderfolgenden Trainingseinheiten korrekt über die vorgegebene Dauer bzw. mit der vorgegebenen Wiederholungszahl durchführt wurde. Das ist der Fall, wenn der Trainer dem Spieler keine oder nur noch wenige Korrekturen geben muss und die Übungsausführung sicher beherrscht wird. Die Zeit bis zum Erreichen des nächsten Levels kann von Spieler zu Spieler und von Übung zu Übung stark variieren. Die Levels sollen nacheinander absolviert und nicht übersprungen werden. Es ist durchaus möglich (und erwünscht), dass sich ein Spieler bei einer Übung auf Level 1 befindet und bei einer anderen Übung bereits das Level 3 beherrscht. Mit FIFA 11+ Kids kann individuell reagiert und trainiert werden.

Organisation auf dem Platz

Die Durchführung des Programms dauert ca. 15 Minuten. Als Hilfsmittel wird ein Ball pro Spieler benötigt. Je nach Leistungsniveau, Alter und Mannschaftsgrösse variiert der Platzbedarf zwischen der Strafraumgrösse bzw. der Hälfte eines Fussballfelds. Der Trainer soll so stehen, dass er alle Spieler sehen kann und auch von allen Spielern gesehen wird.

In der Abbildung ist die Position des Trainers (T) und der Spieler (S) dargestellt. Die weissen Pfeile zeigen die Bewegungsrichtung der Spieler zur gegenüberliegenden Linie. Es ist auf genügend Abstand zwischen den Spielern zu achten, insbesondere bei den Sprung- und Fallübungen.

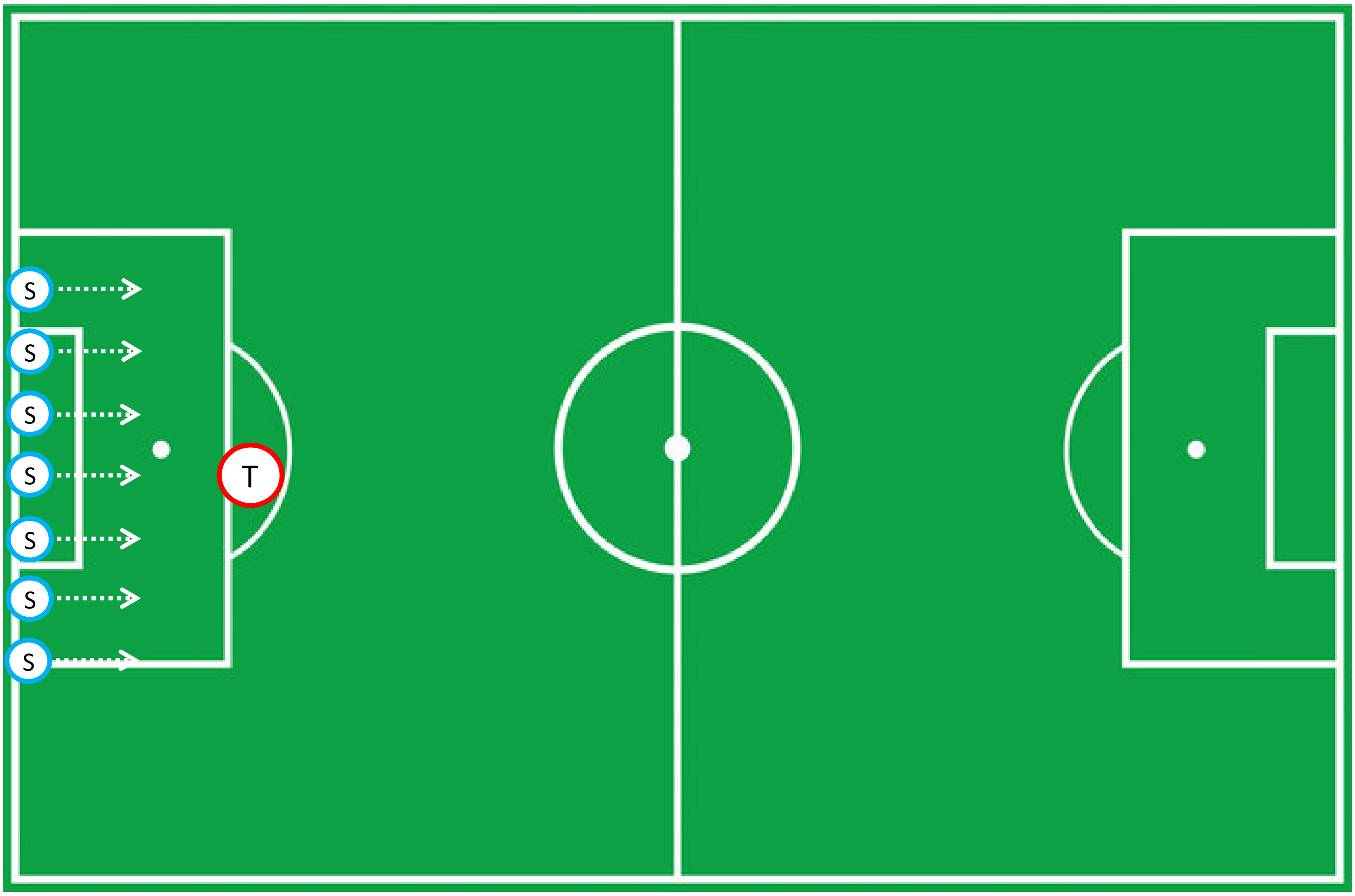

Korrekte Ausführung und Kernelemente

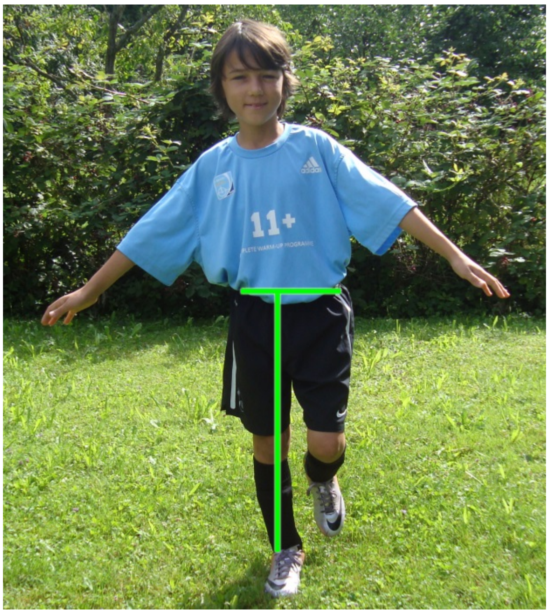

Gerade Beinachse und stabiler, aufrechter Oberkörper

- Fuss, Knie und Hüfte des Standbeins bilden von vorne betrachtet eine gerade Linie
- Fussspitze des Standbeins zeigt nach vorne
- Oberkörper ist aufrecht und zentral positioniert
- Linke und rechte Hüfte sind auf einer Höhe

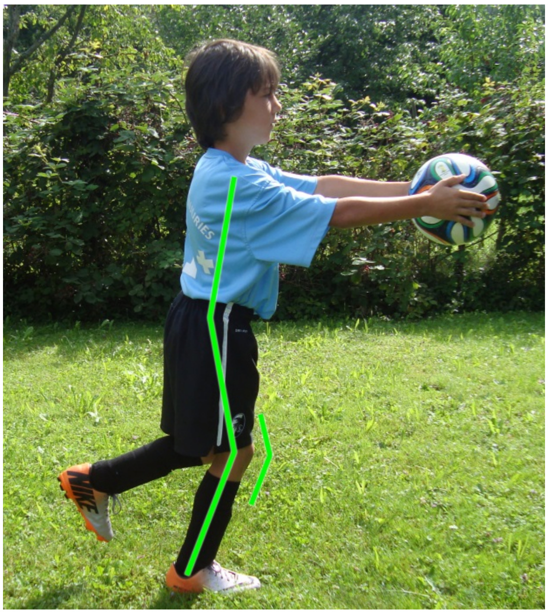

Gebeugtes Knie im Einbeinstand und bei der Landung

- Fussspitze des Standbeins zeigt nach vorne
- Das Knie des Standbeins ist im Stand leicht gebeugt bzw. wird bei der Landung zum Abfedern gebeugt
- Oberkörper ist aufrecht und zentral positioniert
- Der Kopf ist in Neutralstellung

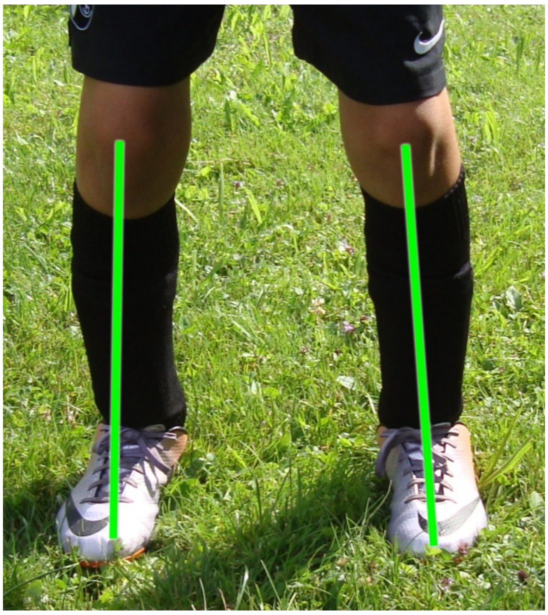

Knie in einer Linie mit der Fussspitze

- Fussspitze und Knie befinden sich in einer Linie
- Auch bei gebeugtem Knie zeigen Knie und Fussspitze in die selbe Richtung

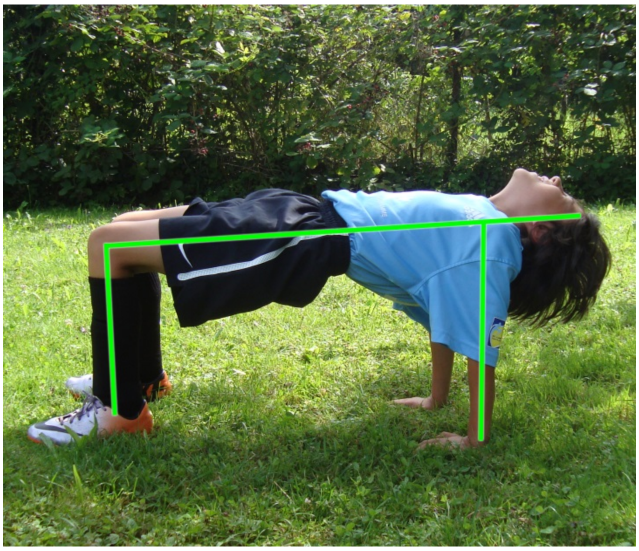

Körperspannung in der „Spider“-Position

- Kopf, Oberkörper und Oberschenkel sind in einer möglichst geraden Line
- Gesäss und Rückenmuskulatur sind angespannt
- Die Spannung wird auch bei der Vorwärtsbewegung gehalten
- Hände sind etwa schulterbreit auseinander

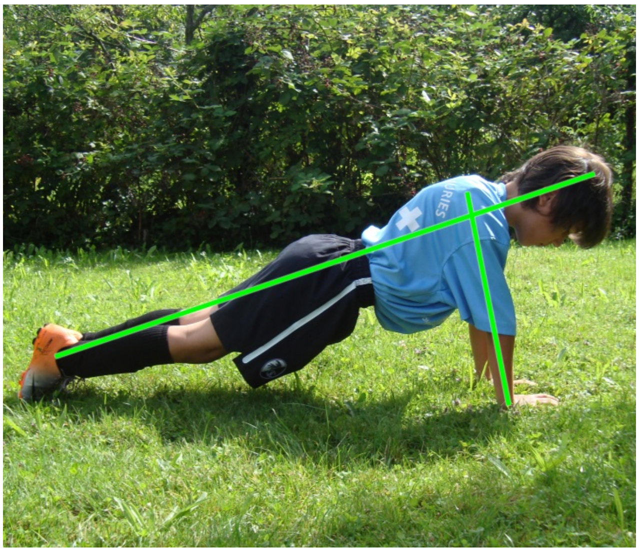

Körperspannung in der Liegestütz-Position

- Kopf, Oberkörper und Beine sind in einer möglichst geraden Line
- Die Bauchmuskulatur ist angespannt
- Der Kopf ist in Neutralstellung
- Hände und Füsse etwa schulterbreit auseinander

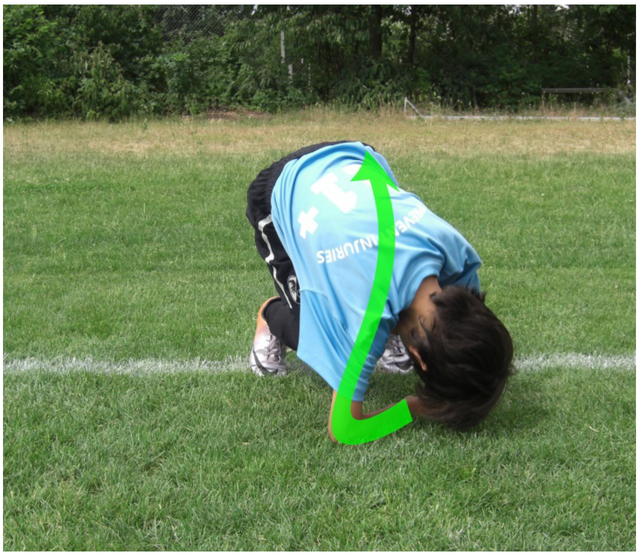

Abrollen über den Rücken

- Die Arme sind beim Bodenkontakt leicht gebeugt
- Abgerollt wird über den vorderen Arm
- Der Kopf berührt nie den Boden
- Abrollbewegung erfolgt diagonal über den Rücken

Für alle Levels gilt:

Fokus: Verbesserung von Gleichgewicht und Koordination.
Ziel: Nach jedem Stopp-Kommando 3 Sekunden stabil auf einem Bein stehen.
Anweisung an die Spieler: „Laufe schnell, bremse ab und stehe stabil auf einem Bein bis zum nächsten Kommando!“

Level 1: Stopp-Kommando hören

Ausgangsposition: Die Spieler stehen an der Grundlinie mit ca. 2 m Abstand zu den Mitspielern. Der Trainer steht etwas ausserhalb des Strafraums (die Distanz altersgemäss anpassen).

Aktion: Auf Kommando des Trainers laufen die Spieler geradeaus in Richtung des Trainers. Wenn der Trainer „rechts“ bzw. „links“ ruft, sollen die Kinder sofort auf dem rechten bzw. linken Bein stehen bleiben und auf einem Bein stehend das Gleichgewicht ca. 3 Sekunden halten. Zwischenhüpfer sind erlaubt, um das Gleichgewicht zu finden. Das Knie des Standbeins soll dabei nicht ganz durchgestreckt sein. Die Arme können zum Ausbalancieren genutzt werden. Wenn ein Spieler nach dem Stopp-Kommando nicht auf einem Bein steht oder innerhalb von 3 Sekunden mit dem anderen Fuss den Boden berührt, so muss er zurück zur Grundlinie joggen. Der Trainer gibt dann erneut das Kommando zum Loslaufen und zum Stoppen (insgesamt pro Durchgang 5 Mal). Dann joggen alle Spieler gemeinsam zurück zur Grundlinie.

Wiederholungen: 3 Durchgänge mit jeweils 5 Stopp-Kommandos.

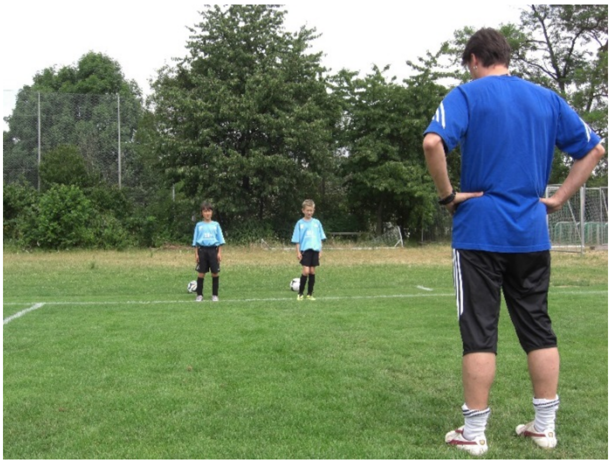

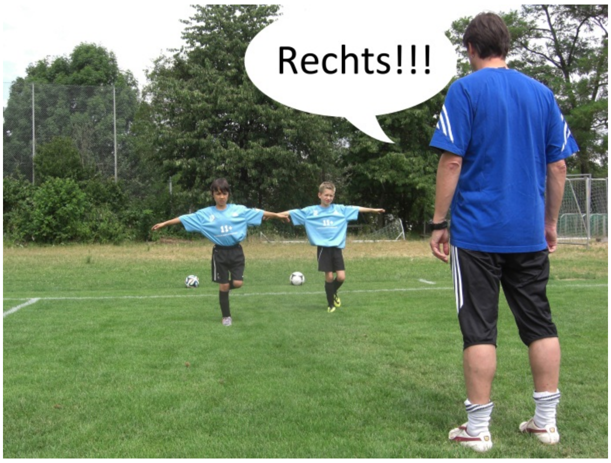

Level 2: Stopp-Kommando sehen

Ausgangsposition und Aktion: Wie Level 1, jedoch sollen die Kinder den Trainer beobachten und das Stopp-Kommando sehen. Der Trainer zeigt an, ob die Kinder auf dem rechten oder linken Bein stoppen sollen.

Wiederholungen: 3 Durchgänge mit jeweils 5 Stopp-Kommandos.

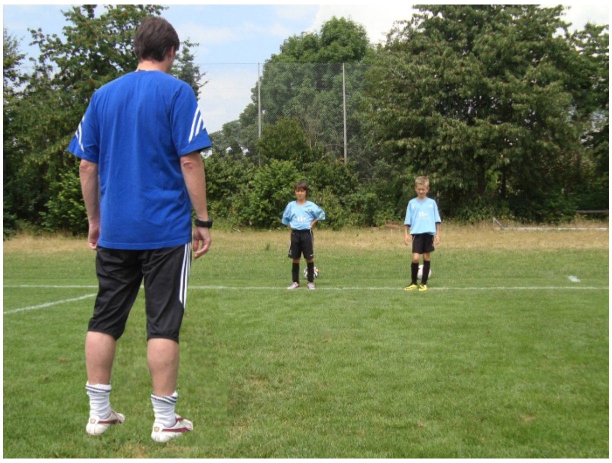

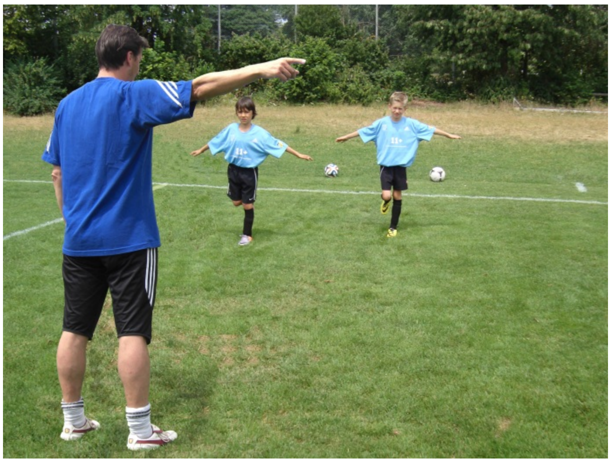

Level 3: Ball in den Händen und Stopp-Kommando hören

Ausgangsposition und Aktion: Wie Level 1, zusätzlich halten die Spieler bei der Übung einen Ball mit beiden Händen.

Wiederholungen: 3 Durchgänge mit jeweils 5 Stopp-Kommandos.

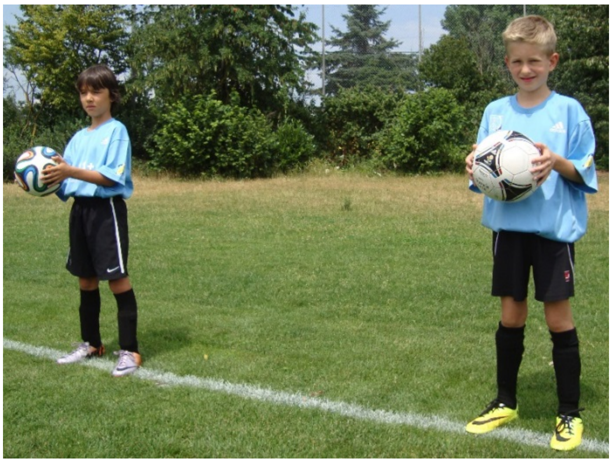

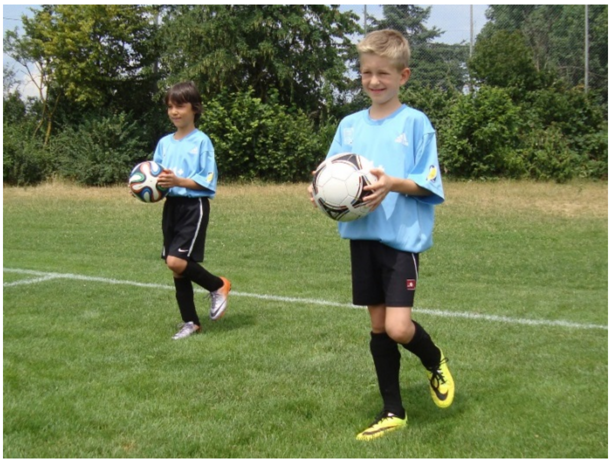

Level 4: Ball in den Händen und Stopp-Kommando sehen

Ausgangsposition und Aktion: Wie Level 2, zusätzlich halten die Spieler bei der Übung einen Ball mit beiden Händen.

Wiederholungen: 3 Durchgänge mit jeweils 5 Stopp-Kommandos.

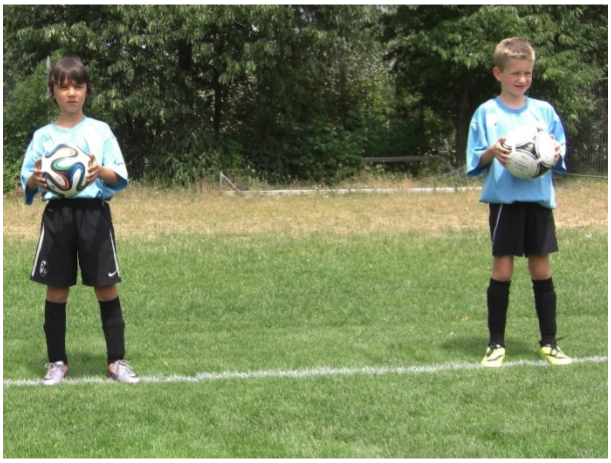

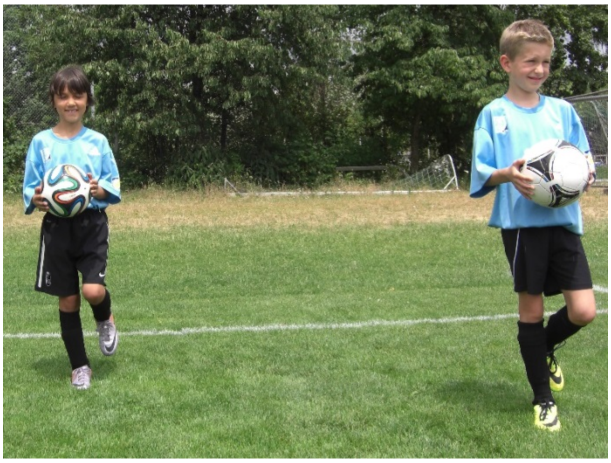

Level 5: Ball dribbeln und Stopp-Kommando hören

Ausgangsposition und Aktion: Wie Level 1, zusätzlich dribbeln die Kinder mit dem Ball am Fuss. Beim Stopp-Kommando stoppen sie den Ball kurz mit dem Fuss ab und bleiben dann auf einem Bein stehen, wobei der freie Fuss den Ball nicht berührt.

Wiederholungen: 3 Durchgänge mit jeweils 5 Stopp-Kommandos.

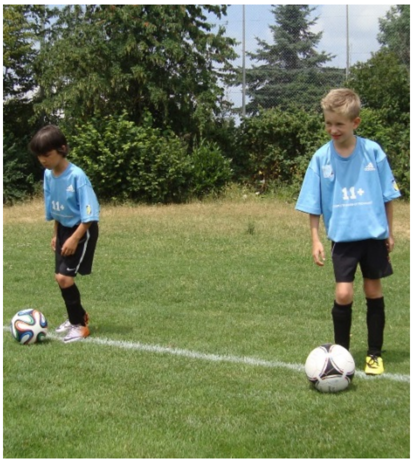

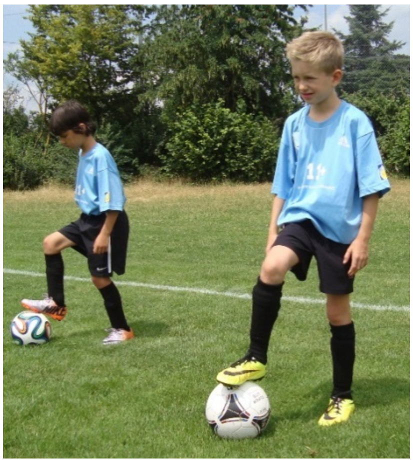

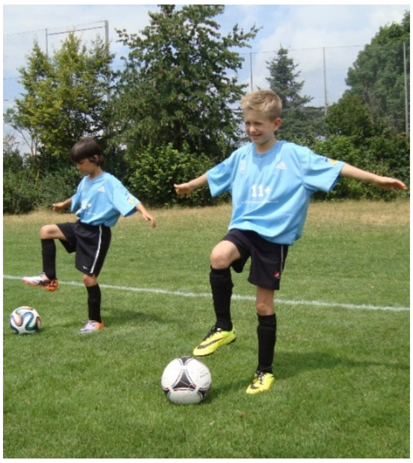

Übung 1: Laufspiel „Wachmann“

Wichtig und richtig:

- ✓ Hüfte, Knie und Fuss sollen von vorne gesehen eine gerade Linie bilden.
- ✓ Das Knie des Standbeins soll leicht gebeugt sein.
- ✓ Fuss gerade aufsetzen, Fussspitze soll nach vorne zeigen.
- ➢ Laufstrecke altersgerecht anpassen.

Diese Fehler bitte unbedingt korrigieren:

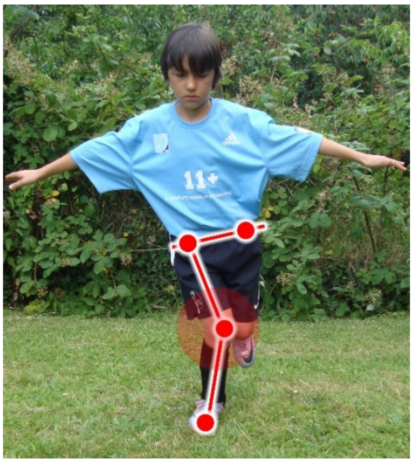

Fehler: Knie-Knick und schiefes Becken

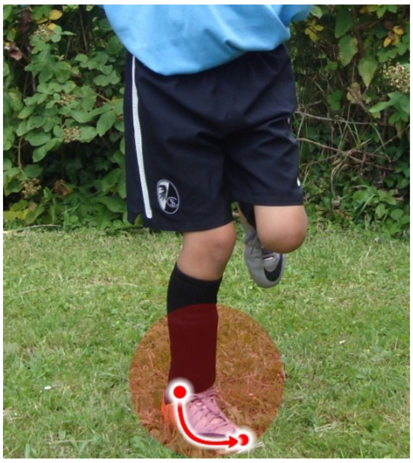

Fehler: Fussdrehung nach innen

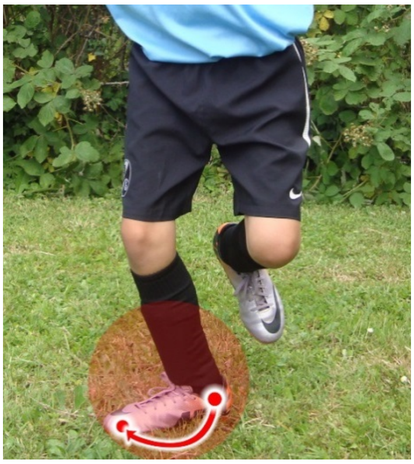

Fehler: Fussdrehung nach aussen

Für alle Levels gilt:

Fokus: Stabilisation von Fuss- und Kniegelenk.
Ziel: Nach jeder Landung das Gleichgewicht finden und 3 Sekunden auf einem Bein stehen.
Anweisung an die Spieler: „Springe weit, lande sicher und stehe stabil bis zum nächsten Sprung!"

Level 1: Landen lernen

Ausgangsposition: Die Spieler stehen an der Grundlinie mit ca. 2 m Abstand zu den Mitspielern. Der Trainer sagt an, auf welchem Bein die Spieler stehen sollen und achtet darauf, dass alle Spieler auf dem gleichen Bein stehen.

Aktion: Auf Kommando des Trainers („und hopp") springen die Spieler einbeinig nach *schräg vorne* und landen auf dem *anderen* Bein. Der Trainer zeigt die Richtung an, um Kollisionen zu vermeiden. Beispiel: Erfolgt der Absprung vom linken Bein, so springen die Spieler nach rechts vorne. Nach der Landung sollen die Spieler das Gleichgewicht finden und ca. 3 Sekunden auf einem Bein stehen bleiben. Während des Balancierens ist das Knie des Standbeins leicht gebeugt. Das Ausbalancieren mit den Armen ist erlaubt. Dann gibt der Trainer das Kommando zum nächsten Sprung in die andere Richtung. Bei jedem Sprung soll eine deutliche Seitwärtsbewegung erzielt werden, sodass sich die Spieler im Zick-Zack vorwärts bewegen. Nach 5 Sprüngen auf jedem Bein gehen die Spieler langsam zur Grundlinie zurück.

Wiederholungen: 2 Durchgänge mit jeweils 10 Sprüngen (5 pro Bein).

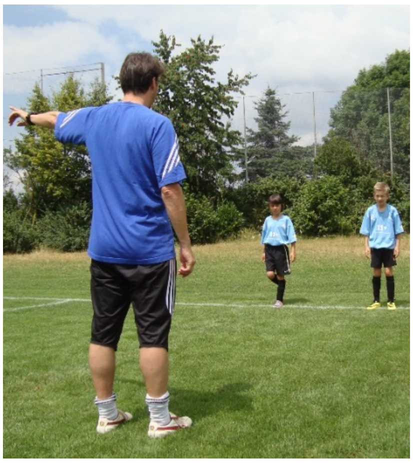
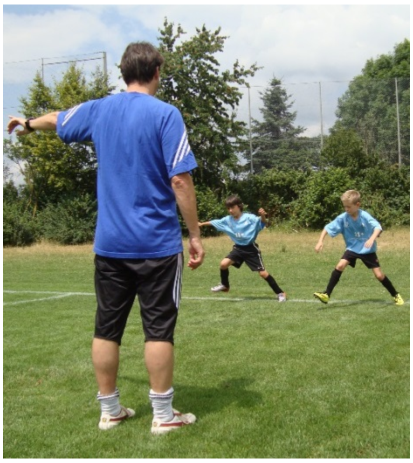
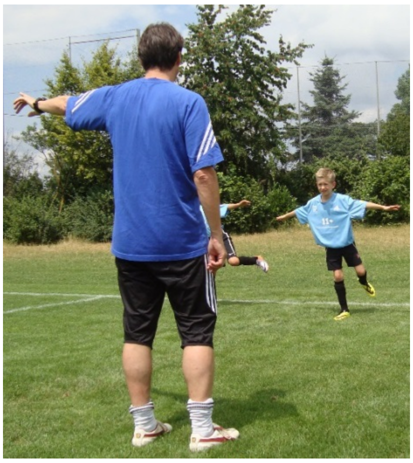

Level 2: Ball in beiden Händen

Ausgangsposition und Aktion: Wie Level 1, zusätzlich halten die Spieler bei der Übung einen Ball mit beiden Händen.

Wiederholungen: 2 Durchgänge mit jeweils 10 Sprüngen (5 pro Bein).

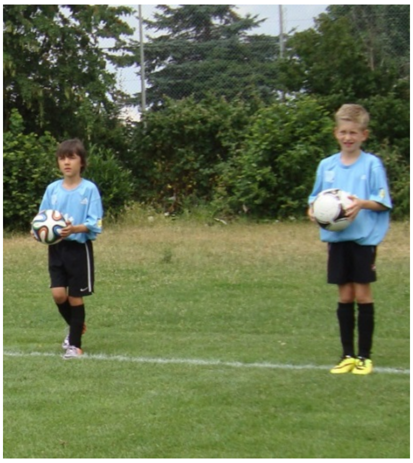
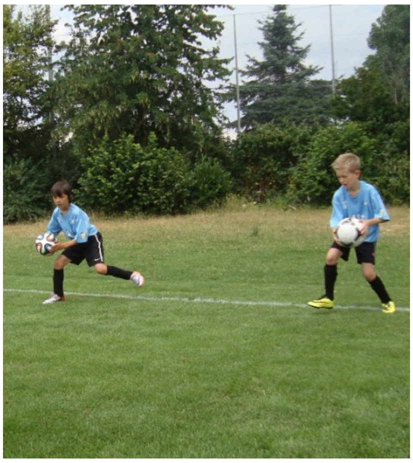
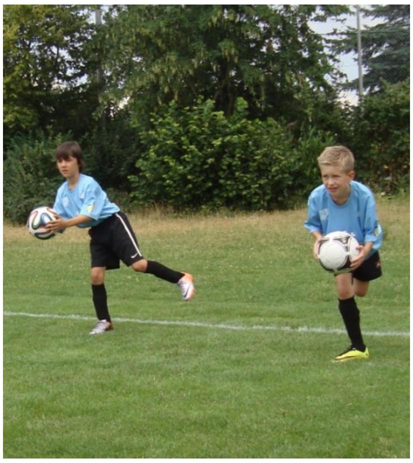

Level 3: Ball in einer Hand balancieren

Ausgangsposition und Aktion: Wie Level 1, zusätzlich balancieren die Spieler den Ball auf der offenen Handfläche (ein Durchgang auf der rechten und ein Durchgang auf der linken Hand).

Wiederholungen: 2 Durchgänge mit jeweils 10 Sprüngen (5 pro Bein).

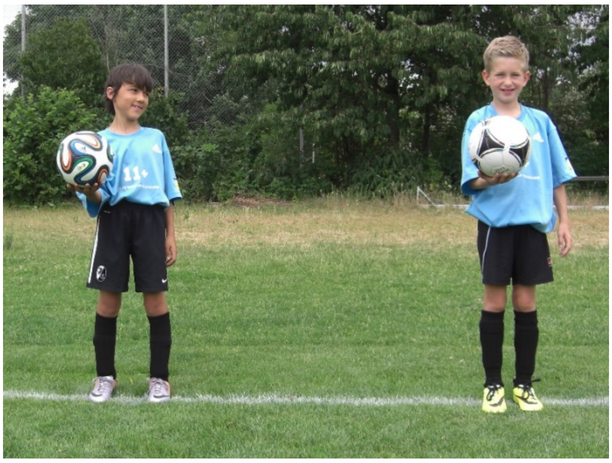
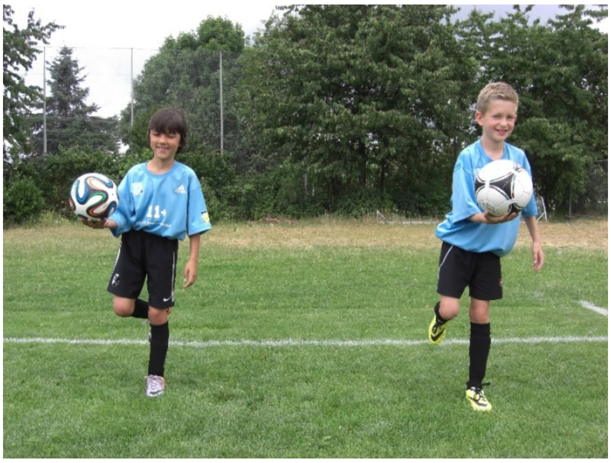

Level 4: Ball auf den Boden tippen

Ausgangsposition und Aktion: Wie Level 2, zusätzlich strecken die Spieler nach der Landung, während sie auf einem Bein stehen, den Ball über den Kopf, beugen sich dann nach vorne, tippen den Ball kurz auf den Boden und richten sich anschliessend gleich wieder auf. Dies soll langsam und kontrolliert geschehen.

Wiederholungen: 2 Durchgänge mit jeweils 10 Sprüngen (5 pro Bein).

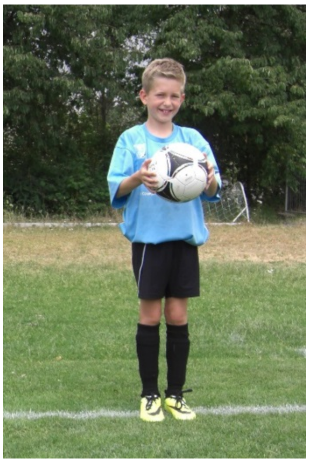

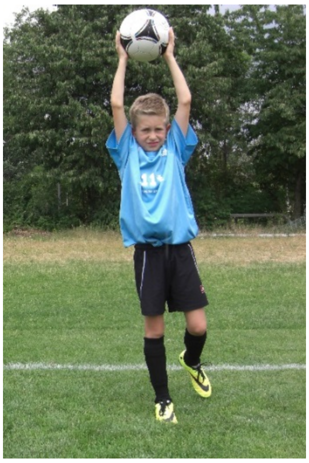

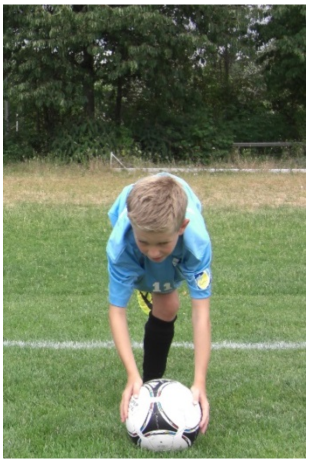

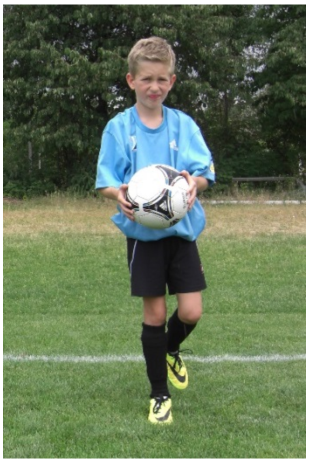

Level 5: Dynamische Standwaage mit Ball

Ausgangsposition und Aktion: Wie Level 4, zusätzlich strecken die Spieler nach der Landung das freie Bein nach hinten-oben und beide Arme nach vorne aus und kehren danach zurück in den normalen Einbeinstand. Anschliessend erfolgt der nächste Sprung. Die Spieler sollen sich beim Strecken „möglichst lang machen". Ideal ist es, wenn Ball, Kopf, Rumpf und das angehobene Bein eine waagerechte Linie bilden (parallel zum Boden).

Wiederholungen: 2 Durchgänge mit jeweils 10 Sprüngen (5 pro Bein).

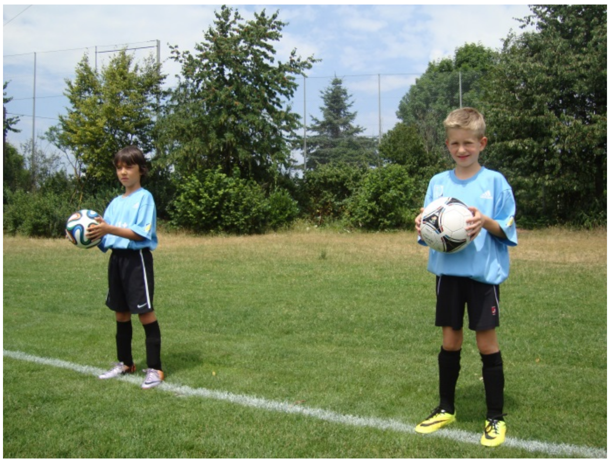

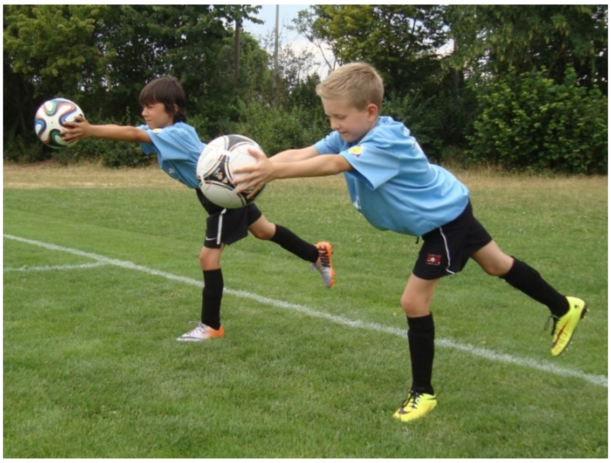

Wichtig und richtig:

- ✓ Hüfte, Knie und Fuss des Sprungbeins sollen von vorne gesehen eine gerade Linie bilden.
- ✓ Hüfte und Knie des Standbeins immer leicht gebeugt halten.
- ✓ Mit gebeugtem Knie weich landen und abfedern.
- ✓ Spieler sollen nach jeder Landung das Gleichgewicht finden.
- ✓ Körperspannung: Bauch- und Rückenmuskulatur sind angespannt. Der Rücken ist gerade und der Kopf in Verlängerung der Wirbelsäule
- ➢ Sprungrichtung anzeigen, damit alle in dieselbe Richtung springen.

Diese Fehler bitte unbedingt korrigieren:

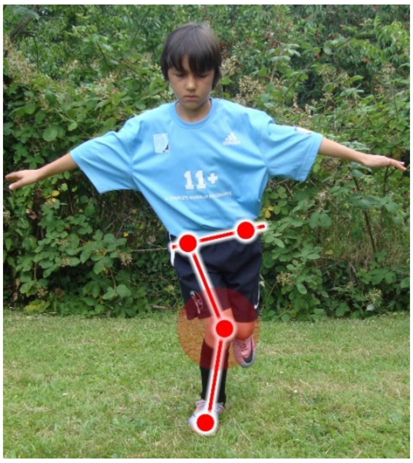

Fehler: Knieknick und schiefes Becken

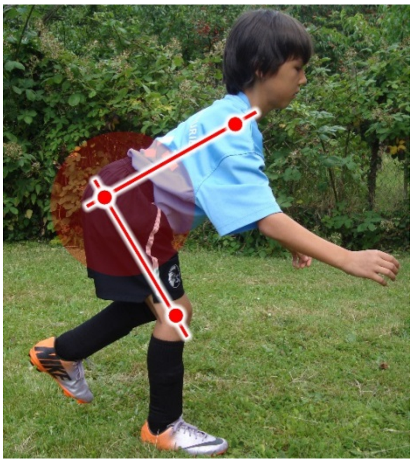

Fehler: Starke Hüftbeugung, nach vorne Überlehnen

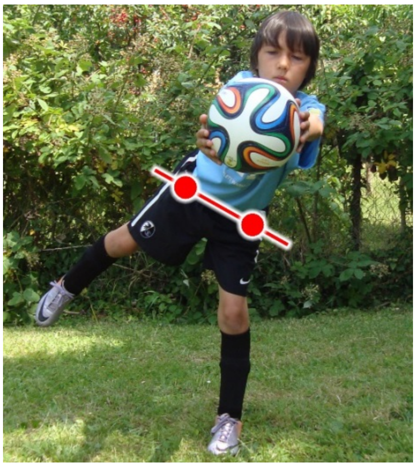

Fehler: Öffnen des Beckens, Rumpf zur Seite geneigt

Für alle Levels gilt:

Fokus: Gleichgewicht bei Zusatz-Aufgaben.

Ziel: Auch in schwierigen Situationen stabil auf einem Bein stehen können.

Anweisung an die Spieler: „Behaltet das Gleichgewicht auch in schwierigen Situationen!“

Level 1: Ball zuwerfen

Ausgangsposition: Zwei Spieler stehen sich mit ca. 3-5 m Abstand im Einbeinstand gegenüber.

Aktion: Die Spieler werfen sich den Ball abwechselnd zu. Anfangs sollte der Abstand verkürzt werden und eine einfache Wurftechnik verwendet werden. Wurfvarianten (beidhändig, einhändig, Druckpass usw.) sind später denkbar.

Wiederholungen: 1 Durchgang auf jedem Bein mit je 5 Würfen pro Spieler.

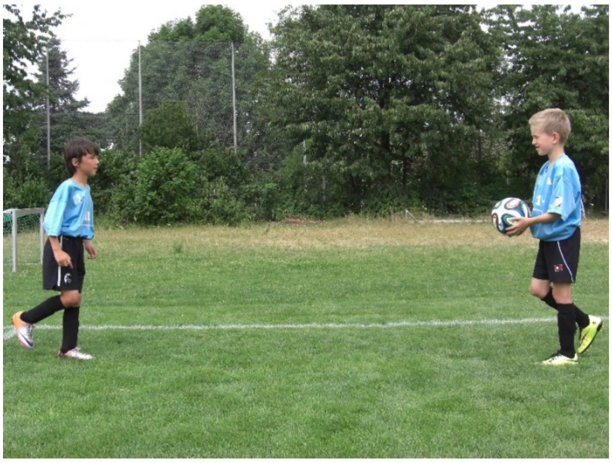

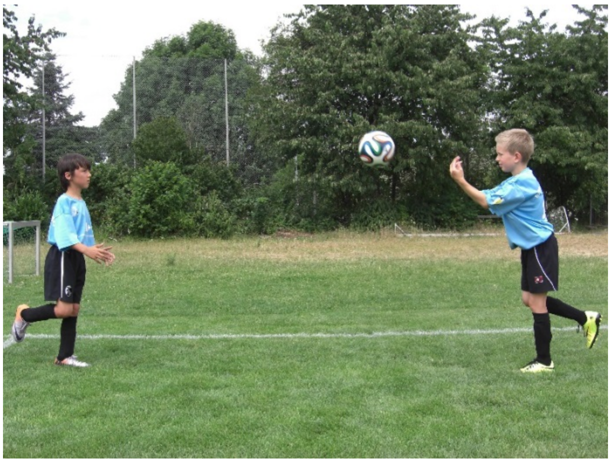

Level 2: Ball zuwerfen und um das angehobene Spielbein kreisen

Ausgangsposition und Aktion: Wie Level 1, zusätzlich kreist der Spieler den Ball nach dem Fangen um das angehobene Spielbein.

Wiederholungen: 1 Durchgang auf jedem Bein mit je 5 Würfen pro Spieler.

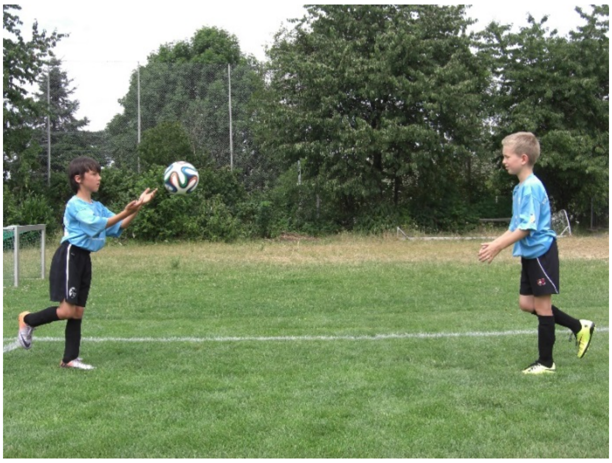

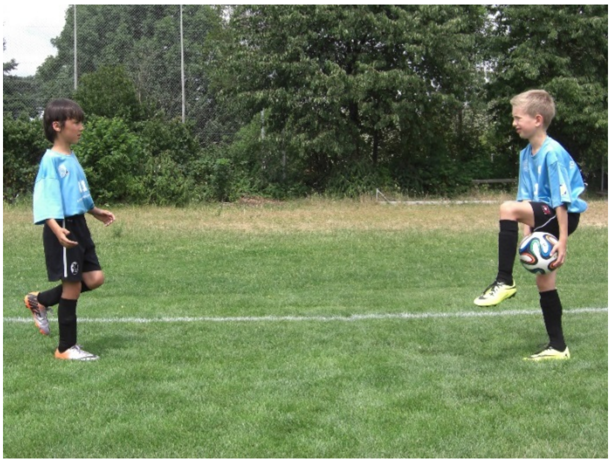

Level 3: Passspiel

Ausgangsposition: Zwei Spieler stehen sich mit ca. 2-5 m Abstand im Einbeinstand gegenüber.

Aktion: Die Spieler passen sich den Ball flach mit der Fussinnenseite zu. Vor dem Zurückpassen sollte der Ball zuerst gestoppt werden. Die Pässe sollen so präzise wie möglich erfolgen, sodass die Spieler auf der Stelle stehenbleiben können. Passvarianten sind später denkbar.

Wiederholungen: 1 Durchgang auf jedem Bein mit je 5 Pässen pro Spieler.

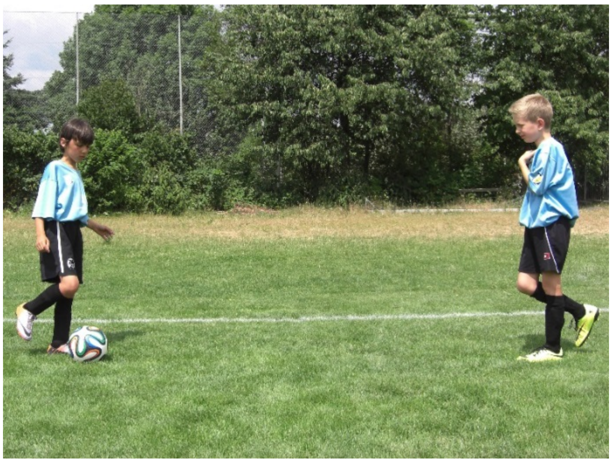

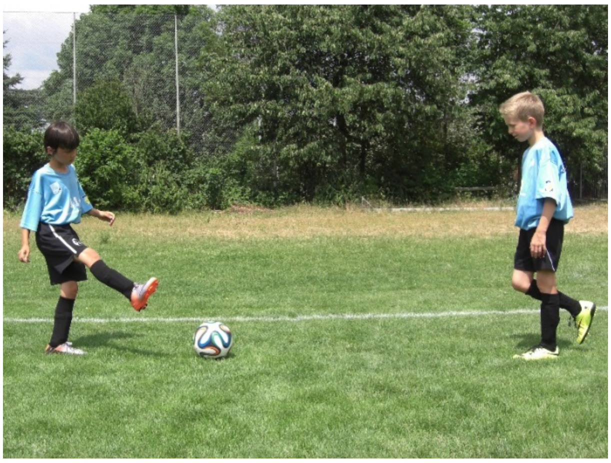

Level 4: Ball zuwerfen und ohne Bodenberührung zurückpassen

Ausgangsposition: Wie Level 3.

Aktion: Ein Spieler wirft dem Partner den Ball so zu, dass dieser den Ball mit dem Fuss zurückpassen kann. Der Pass soll volley aus der Luft (d.h. ohne vorherigen Bodenkontakt) und so präzise wie möglich gespielt werden, sodass der Spieler, der den Ball geworfen hat, den Ball auch wieder fangen kann. Damit der Spieler korrekt zurück passen kann ist es wichtig, dass der Partner präzise wirft. Zwischenhüpfer sind erlaubt.

Wiederholungen: 1 Durchgang auf jedem Bein mit je 5 Würfen pro Spieler.

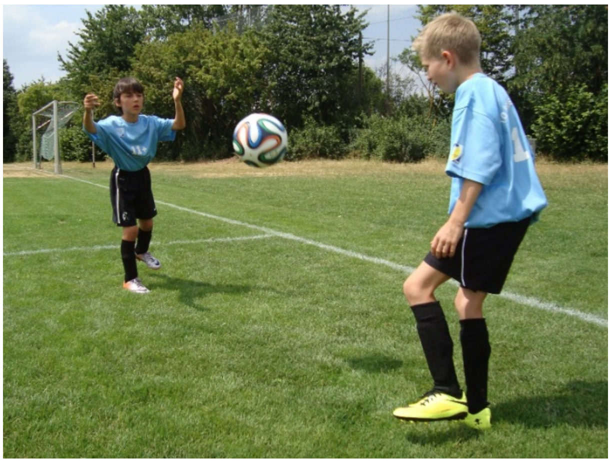

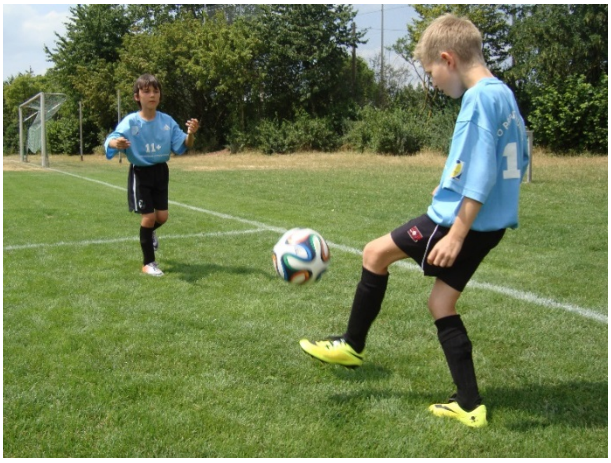

Level 5: Gleichgewicht des Partners testen

Ausgangsposition: Zwei Spieler stehen sich im Einbeinstand in Reichweite gegenüber. Beide halten jeweils einen Ball frei vor dem Körper in beiden Händen.

Aktion: Nun drücken sie die Bälle auf Brusthöhe gegeneinander und versuchen, sich gegenseitig aus dem Gleichgewicht zu bringen. Berührt ein Spieler mit dem angehobenen Fuss (Spielbein) den Boden, wird neu begonnen. Die Spieler dürfen auf einem Bein hüpfen, um das Gleichgewicht zu halten. Es dürfen keine schlagenden Bewegungen ausgeführt werden, die Bälle müssen ständig im Kontakt bleiben. Der Trainer wählt die Übungspartner in Abhängigkeit von Grösse und Geschicklichkeit aus. Es ist möglich, einen Wettkampf aus der Übung zu machen: Der Spieler, der seinen Partner aus dem Gleichgewicht bringt, bekommt einen Punkt.

Wiederholungen: 1 Durchgang auf jedem Bein für jeweils 20 Sekunden.

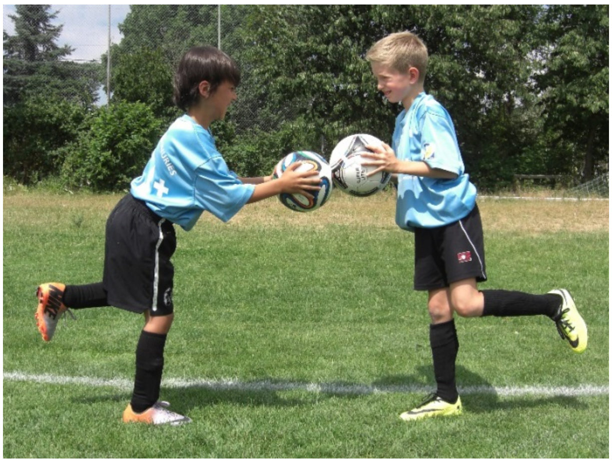

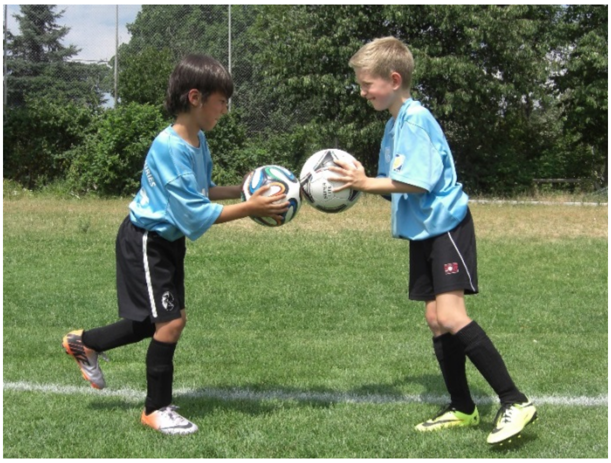

Übung 3: Einbeinstand

Wichtig und richtig:

- ✓ Die Fussspitze des Standbeins zeigt nach vorne.
- ✓ Hüfte, Knie und Fuss des Sprungbeins sollen von vorne gesehen eine gerade Linie bilden.
- ✓ Hüfte und Knie des Standbeins immer leicht gebeugt halten.
- ✓ Die Beckenlinie ist waagerecht.
- ✓ Körperspannung: Bauch- und Rückenmuskulatur sind angespannt. Der Rücken ist gerade und der Kopf in Verlängerung der Wirbelsäule.

Diese Fehler bitte unbedingt korrigieren:

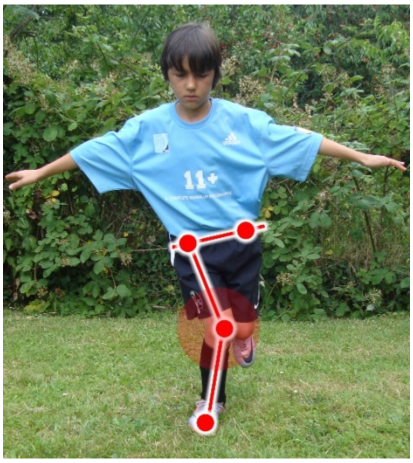

Fehler: „Knieknick“ und schiefes Becken

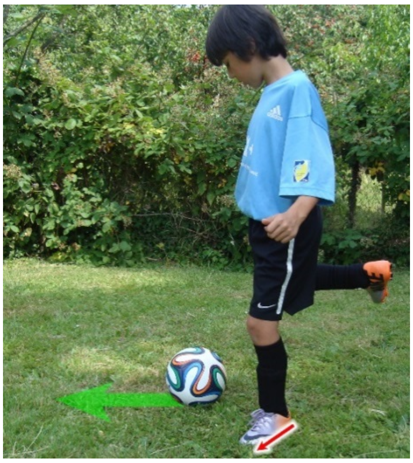

Fehler: Fussspitze des Stand-beines nicht in Passrichtung

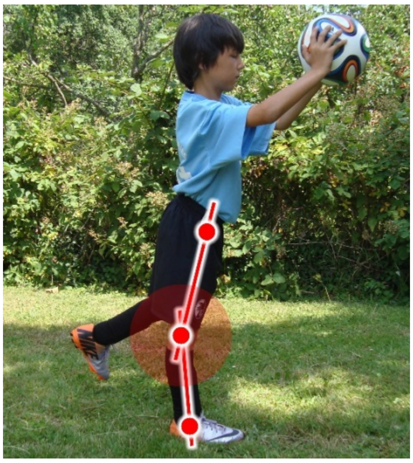

Fehler: Überstrecktes Knie

Übung 4: Liegestütz

Für alle Übungen gilt:

Fokus: Kräftigung der Rumpf- und Armmuskulatur.

Ziel: Die Körperspannung während der Übungen halten können.

Anweisung an die Spieler: „Euer Körper bildet vom Kopf bis zu den Füssen eine möglichst gerade Linie! Spannt Bauch und Rücken an!"

Level 1: Tunnel

Ausgangsposition: Ein Spieler steht, die restlichen Spieler befinden sich dicht nebeneinander in der Liegestützposition und bilden gemeinsam einen „Tunnel". Hände und Füsse sind dabei jeweils etwa hüftbreit auseinander.
Aktion: Der stehende Spieler rollt einen Ball durch den Tunnel. Anschliessend geht er am Anfang des Tunnels in die Liegestütz-Position und erweitert so den Tunnel. Nachdem der Ball unter allen Spielern hindurchgerollt ist, nimmt der letzte Spieler des Tunnels den Ball auf, läuft zum Anfang des Tunnels, rollt den Ball durch den Tunnel und geht am Anfang des Tunnels in die Liegestützposition. Der Tunnel „wandert" dadurch weiter. Die Übung kann als Wettkampf zwischen zwei Gruppen durchgeführt werden.
Wiederholungen: 2 Durchgänge, bei denen jedes Kind jeweils einmal den Ball rollt (maximal 8 Kinder pro Gruppe).

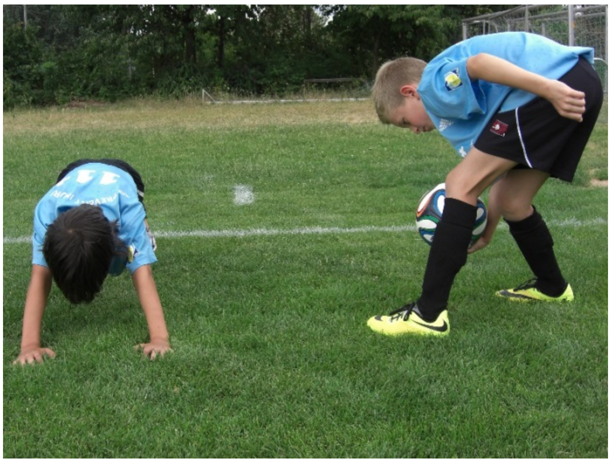

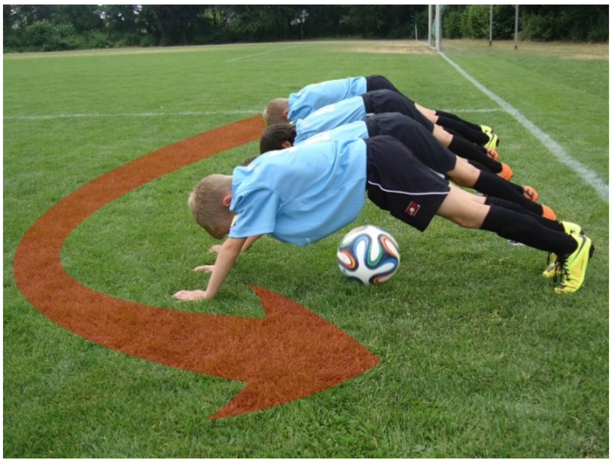

Level 2: Unterarmstütz; Schienbeine liegen auf dem Ball

Ausgangsposition: Die Spieler sind in der Unterarmstützposition. Dabei liegen die Unterarme flach auf dem Boden. Beide Schienbeine liegen mittig auf dem Ball auf und der Blick ist auf den Boden gerichtet. Der Kör-per bildet vom Kopf bis zu den Füßen eine gerade Linie.

Aktion: Zunächst ziehen sich die Spieler auf dem Ball nach vorne, bis der Ball die Füsse leicht berührt und anschliessend schieben sie sich nach hinten, bis der Ball sich fast unter den Knien befindet. Die Bewegungen werden langsam und kotrolliert ausgeführt. Die Unterarme bleiben dabei an derselben Stelle und die Beine immer auf dem Ball.

Wiederholungen: 3 Durchgänge à je 15 Sekunden.

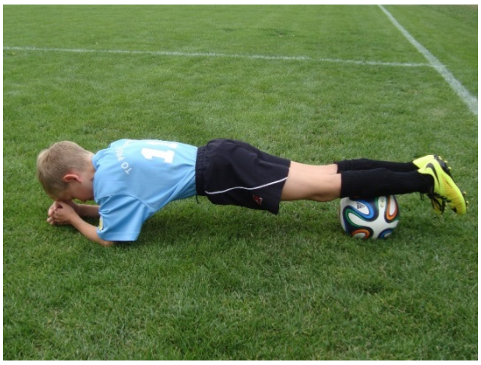
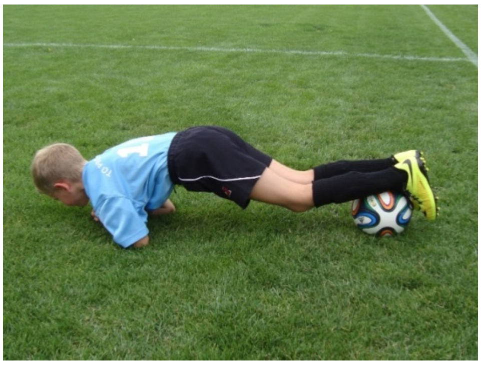
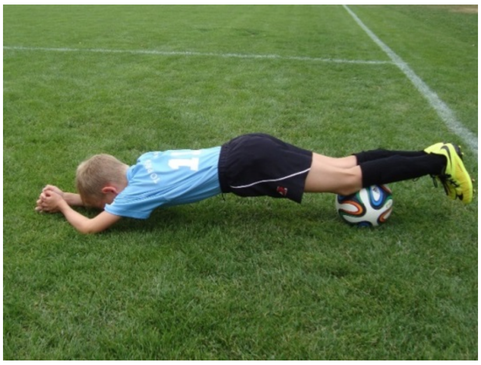

Level 3: Ball um die Hände rollen

Ausgangsposition: Die Spieler sind in der Liegestützposition. Es liegt ein Ball vor jedem Spieler.

Aktion: Sie heben eine Hand vom Boden und rollen mit dieser Hand den Ball um die andere, abgestützte Hand. Dann stützten sie sich mit der zuvor freien Hand ab und rollen den Ball um die andere Hand. So rollen sie den Ball abwechselnd mit beiden Händen in Form einer Acht.

Wiederholungen: 3 Durchgänge à je 15 Sekunden.

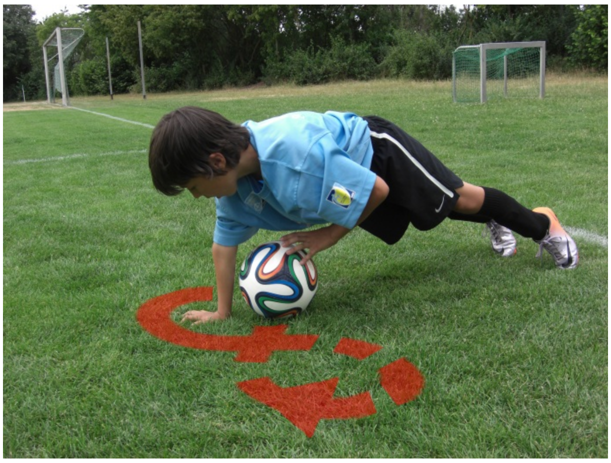
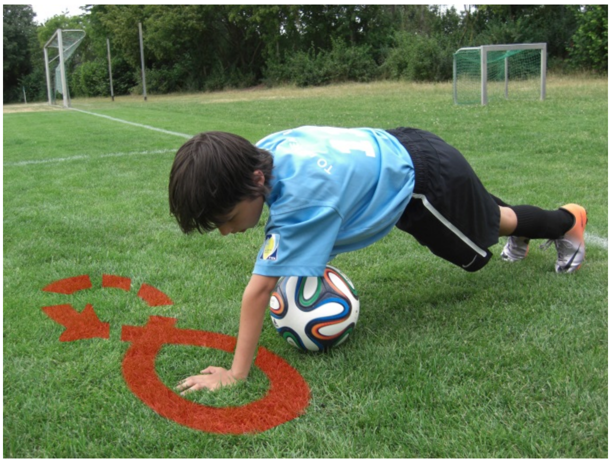

Level 4: Ball zwischen Händen und Füssen rollen

Ausgangsposition: Wie Level 3.

Aktion: Die Spieler rollen den Ball mit der linken Hand unter dem Körper zum linken Fuss. Anschliessend passen sie sich den Ball mit dem linken Fuss zur rechten Hand. Dann wiederholen sie die Übung beginnend mit der rechten Hand (zum rechten Fuss und von dort wieder zur linken Hand) und wechseln so beide Seiten ab.

Wiederholungen: 3 Durchgänge à je 15 Sekunden.

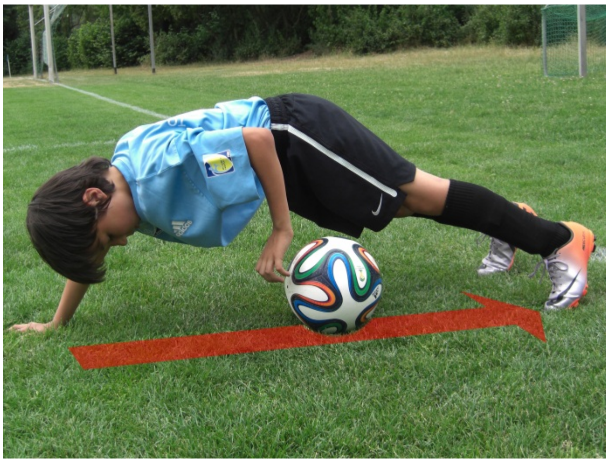

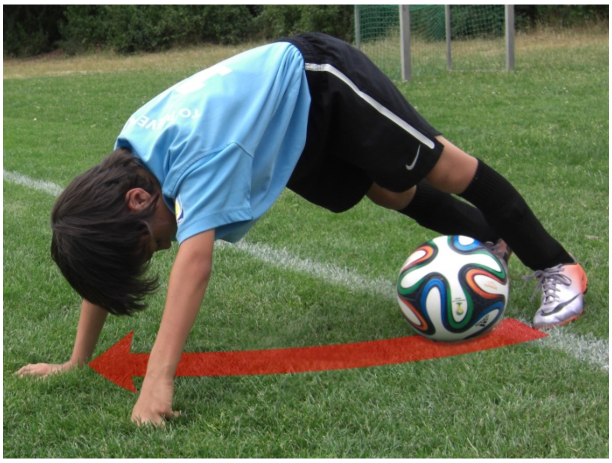

Level 5: Hände auf dem Ball

Ausgangsposition: Wie Level 3, jedoch stützen sich die Spieler mit beiden Händen auf dem Ball ab.

Aktion: Sie gehen langsam mit den Füssen in kleinen Schritten möglichst weit nach hinten und dann wieder nach vorne, in die Ausgangsstellung zurück. Die Bewegungen sollen unbedingt ruhig und kontrolliert ausgeführt werden. Hinweis: Bei feuchtem Rasen auf Level 4 ausweichen.

Wiederholungen: 3 Durchgänge à je 10 Sekunden.

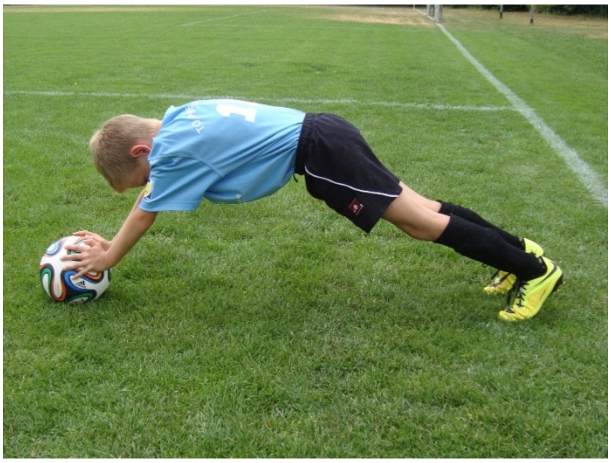

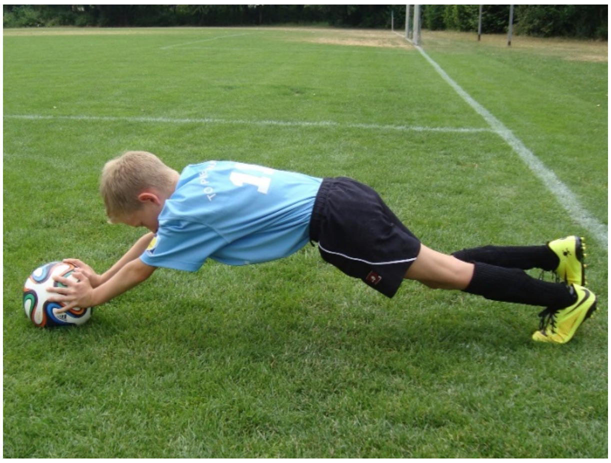

Übung 4: Liegestütz

Wichtig:

- ✓ Kopf, Schultern, Rücken und Becken bilden eine gerade Linie.
- ✓ Bauch und Gesäss anspannen.
- ✓ Blick nach unten auf den Boden.
- ✓ Bewegungen langsam und kontrolliert ausführen.

Diese Fehler bitte unbedingt korrigieren:

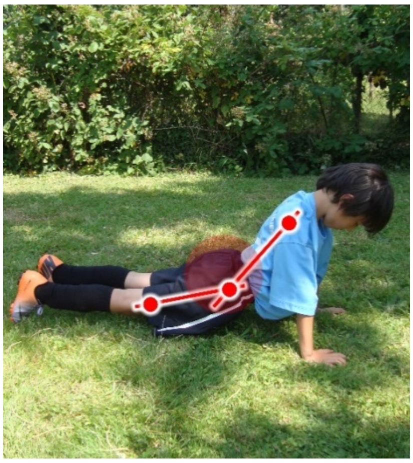

Fehler: Becken zu tief

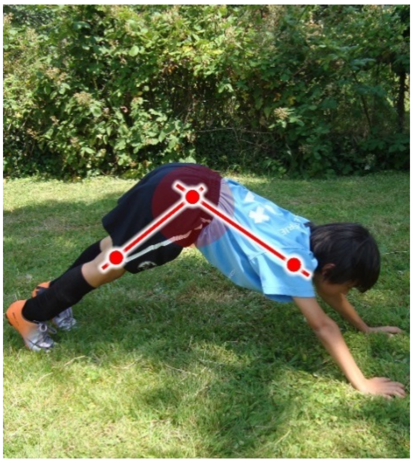

Fehler: Becken zu hoch

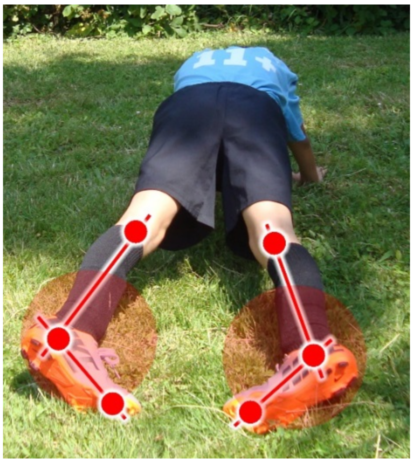

Fehler: Eindrehen der Füsse

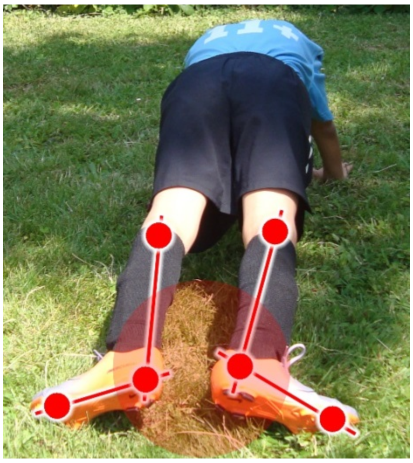

Fehler: Ausdrehen der Füsse

Für alle Übungen gilt:

Fokus: Kräftigung der Beinmuskulatur, Verbesserung von Balance und Koordination.

Ziel: Sicheres, kontrolliertes Landen und weites Springen.

Anweisung an die Spieler: „Springe weit, lande sicher und stehe stabil bis zum nächsten Sprung!“

Level 1: Nach vorn

Ausgangsposition: Die Spieler stehen im Einbandstand an der Grundlinie mit deutlichem Abstand (ca. 2 m) zu den Mitspielern.

Aktion: Auf Kommando des Trainers springen die Spieler einbeinig geradeaus. Der Sprung und die Landung erfolgen auf demselben Bein. Die Sprünge sollen deutlich in die Weite gehen. Nach der Landung erfolgt eine kurze stabile Standphase (ca. 3 Sekunden). Nach einem kompletten Durchgang (insgesamt 10 Sprünge) gehen die Spieler langsam zur Grundlinie zurück.

Wiederholungen: 2 Durchgänge mit je 5 Sprüngen auf dem einen und dann 5 Sprüngen auf dem anderen Bein.

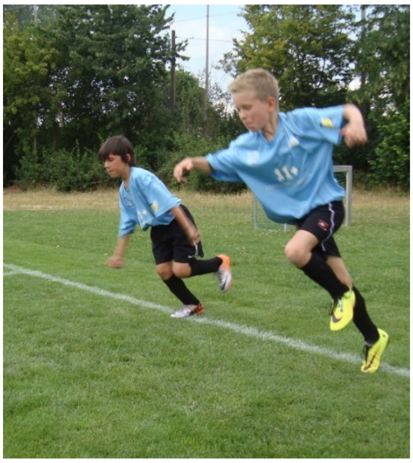
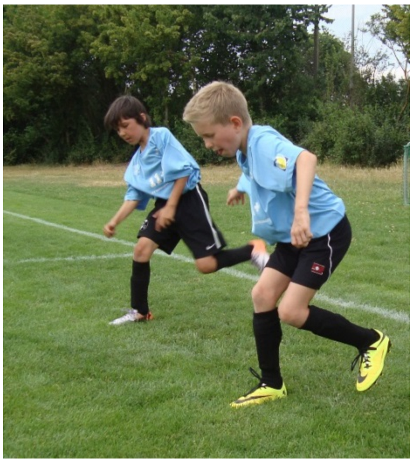
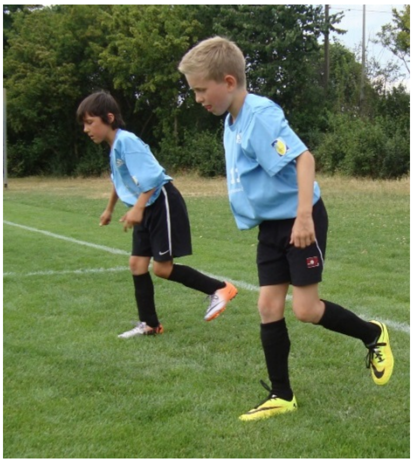

Level 2: Vor und zurück

Ausgangsposition: Die Spieler stehen im Einbandstand ca. 2 m vor der Grundlinie mit deutlichem Abstand (ca. 2 m) zu den Mitspielern.

Aktion: Wie Level 1, jedoch zeigt der Trainer an, ob vorwärts oder rückwärts gesprungen wird.

Wiederholungen: 2 Durchgänge mit je 5 Sprüngen auf dem einen und dann 5 Sprüngen auf dem anderen Bein.

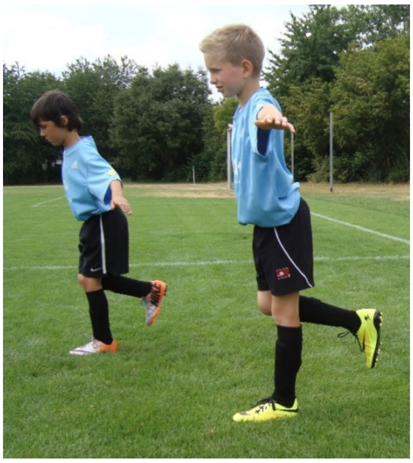

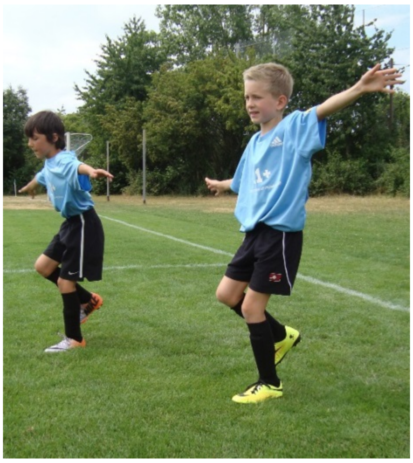

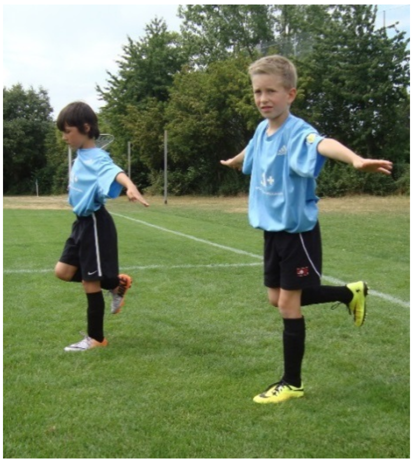

Level 3: Seitwärts

Ausgangsposition und Aktion: Wie Level 2, jedoch springen die Spieler seitwärts. Der Trainer zeigt die Richtung an. Es ist stets auf genügend Abstand zwischen den Spielen zu achten, um Kollisionen zu vermeiden.

Wiederholungen: 2 Durchgänge mit je 5 Sprüngen auf dem einen und dann 5 Sprüngen auf dem anderen Bein.

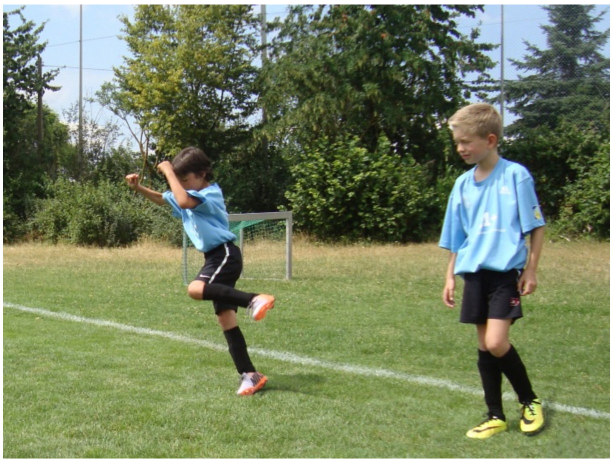

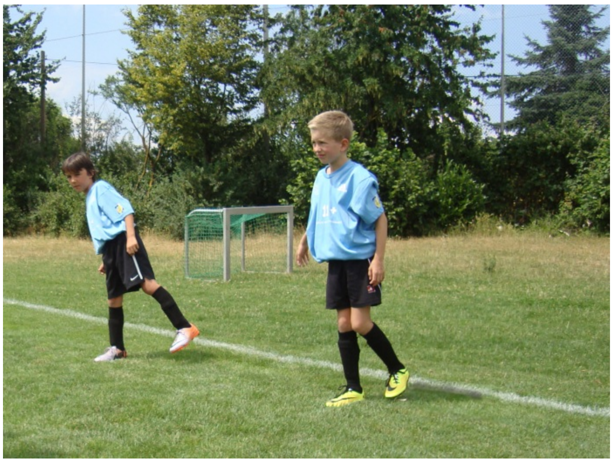

Level 4: Trainer gibt die Richtung an

Ausgangsposition und Aktion: Wie Level 2, jedoch springen die Spieler in die vom Trainer vorgegebene Richtung (nach vorne, nach hinten, nach links, nach rechts). Der Trainer gibt das Kommando („und hopp“) und zeigt gleichzeitig die Richtung an, um Kollisionen zu vermeiden. Auf genügend Abstand zwischen den Spielen achten!

Wiederholungen: 2 Durchgänge mit je 5 Sprüngen auf dem einen und dann 5 Sprüngen auf dem anderen Bein.

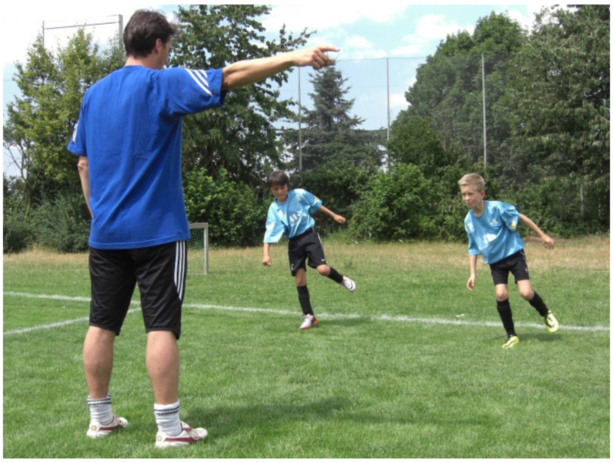

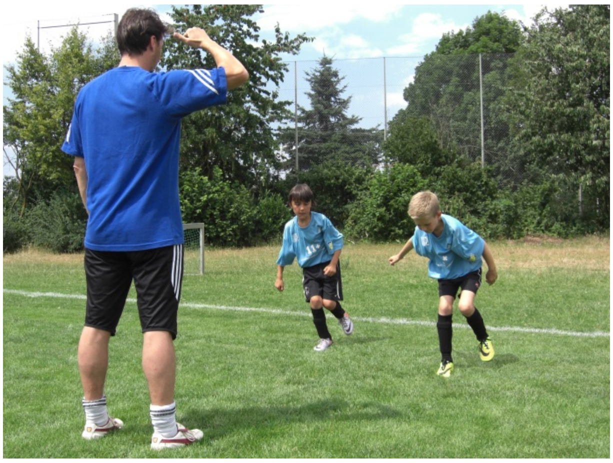

Level 5: Trainer gibt Richtung an; Ball in beiden Händen

Ausgangsposition und Aktion: Wie Level 4, jedoch halten die Spieler einen Ball in den Händen.

Wiederholungen: 2 Durchgänge mit je 5 Sprüngen auf dem einen und dann 5 Sprüngen auf dem anderen Bein.

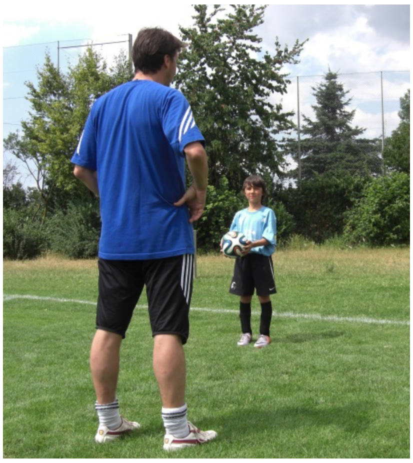

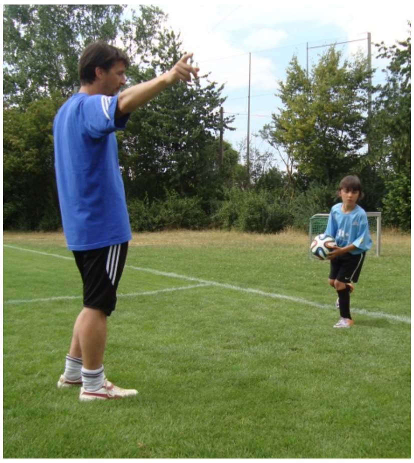

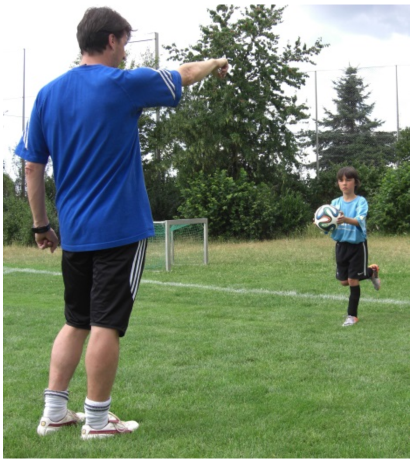

Übung 5: Einbeinsprünge

Wichtig und richtig:

- ✓ Hüfte, Knie und Fuss des Sprungbeins sollen von vorne gesehen eine gerade Linie bilden.
- ✓ Hüfte und Knie des Standbeins immer leicht gebeugt halten.
- ✓ Mit gebeugtem Knie weich auf dem Fussballen landen und abfedern.
- ✓ Spieler sollen nach jeder Landung das Gleichgewicht finden.
- ✓ Auf ausreichend seitlichen Abstand zwischen den Spieler achten!
- ✓ Körperspannung: Bauch- und Rückenmuskulatur sind angespannt. Der Rücken ist gerade und der Kopf in Verlängerung der Wirbelsäule.

Diese Fehler bitte unbedingt korrigieren:

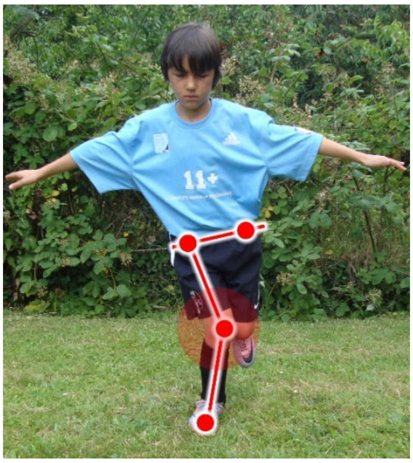

Fehler: Knieknick und schiefes Becken

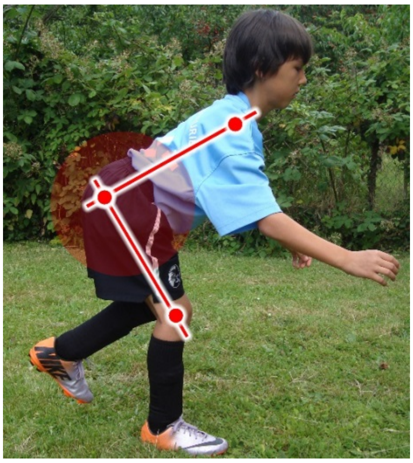

Fehler: Beugung in der Hüfte, starkes Nach-Vorne-Lehnen

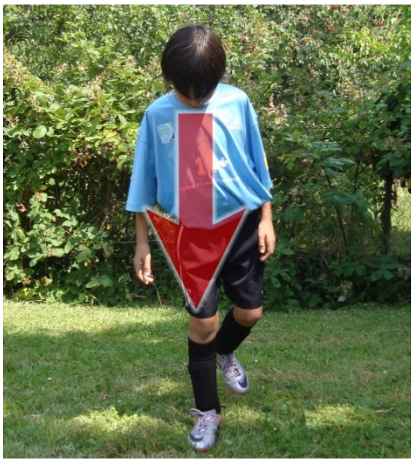

Fehler: Blick zum Boden

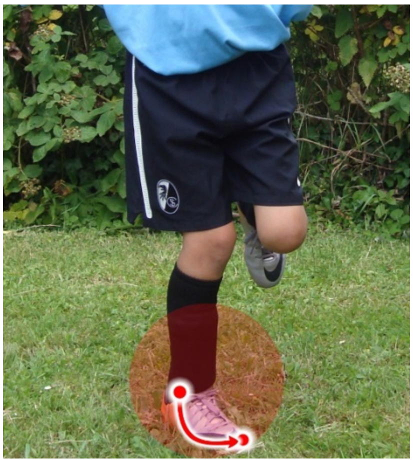

Fehler: Fussdrehung nach innen

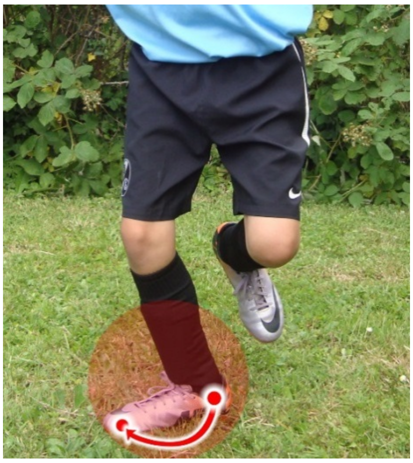

Fehler: Fussdrehung nach aussen

Übung 6: Spiderman

Für alle Übungen gilt:

**Fokus:** Kräftigung der Rumpfmuskulatur und der Oberschenkelrückseite.
**Ziel:** Körperspannung während der gesamten Übung halten können.
**Anweisung an die Spieler:** „Nehmt das Gesäss hoch! Spannt Bauch und Rücken an!“

Level 1: Ball antippen

Ausgangsposition: Die Spieler stützen sich auf Händen und Füssen ab. Hände und Füsse sind dabei jeweils hüftbreit auseinander. Der Rücken zeigt zum Boden. Der Körper bildet eine möglichst gerade Linie vom Kopf bis zu den Knien. Der Ball liegt direkt vor den Füssen.

Aktion: Die Spieler heben ein Bein vom Boden ab, tippen mit dem Fuss auf den Ball und rollen diesen leicht nach vorne und wieder nach hinten. Die Spieler wiederholen die Übung mit dem anderen Bein und führen die Bewegung abwechselnd mit beiden Beinen durch. Die Bewegung soll langsam und kontrolliert ausgeführt werden.

Wiederholungen: 3 Durchgänge à 15 Sekunden.

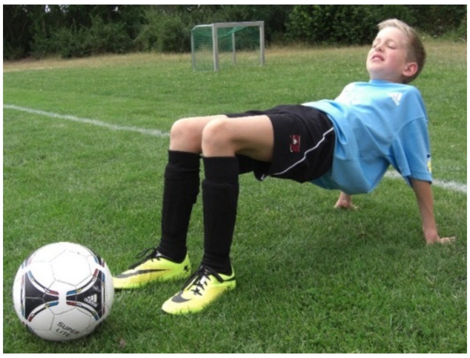
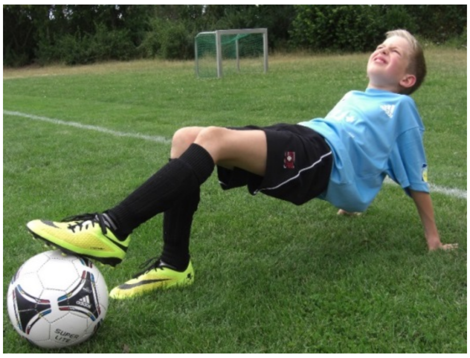
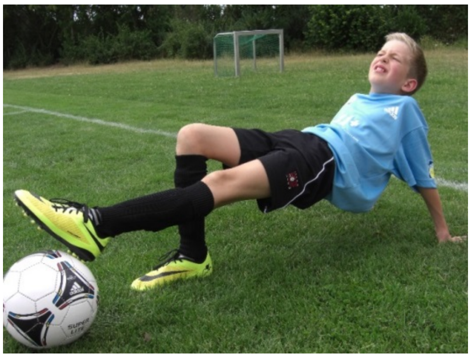

Übung 6: Spiderman

Level 2: Recken und Strecken

Ausgangsposition: Wie Level 1.

Aktion: Aus der Grundposition tasten sich die Spieler zunächst mit den Händen nach hinten und wieder zurück in die Ausgangsstellung (oberes Bilderpaar) und anschliessend mit den Füssen nach vorne bis der Körper möglichst gestreckt ist und wieder zurück (unteres Bilderpaar). Die Spieler tun dies langsam und kontrolliert. Sie wiederholen die Bewegungen immer abwechselnd.

Wiederholungen: 3 Durchgänge à 15 Sekunden.

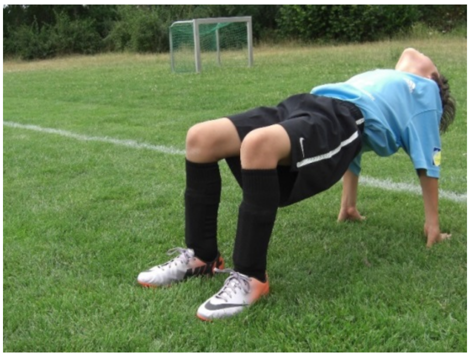
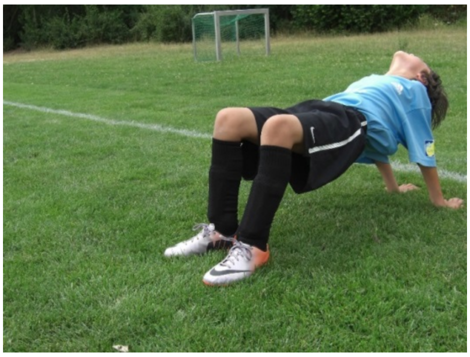
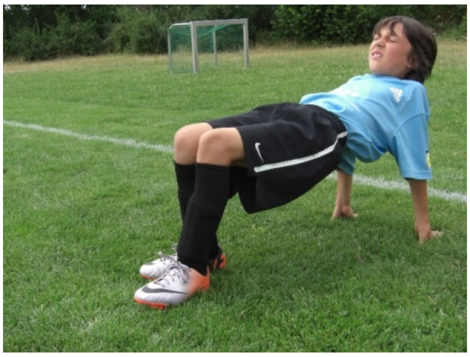
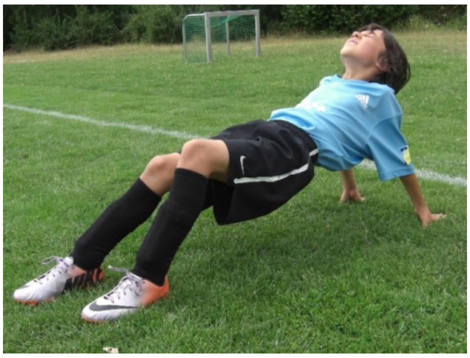

Level 3: Krabbeln

Ausgangsposition: Wie Level 1.

Aktion: Die Spieler bewegen sich auf „allen Vieren" vorwärts (Füsse voraus) in Richtung des Trainers.

Wiederholungen: 3 Durchgänge (je nach Leistungsniveau 5-10 m).

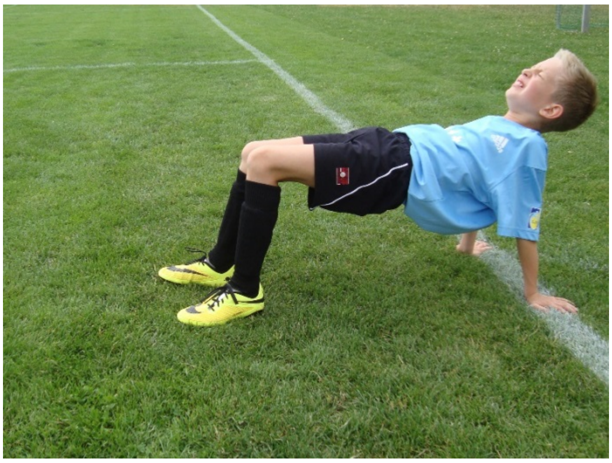
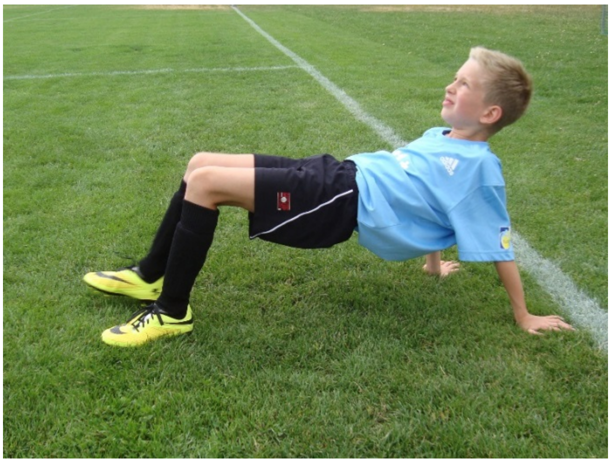

Level 4: Dribbeln

Ausgangsposition und Aktion: Wie Level 3, jedoch „dribbeln“ die Spieler einen Ball. Der Ball soll dabei kontrolliert geführt werden.

Wiederholungen: 3 Durchgänge (je nach Leistungsniveau 5-10 m).

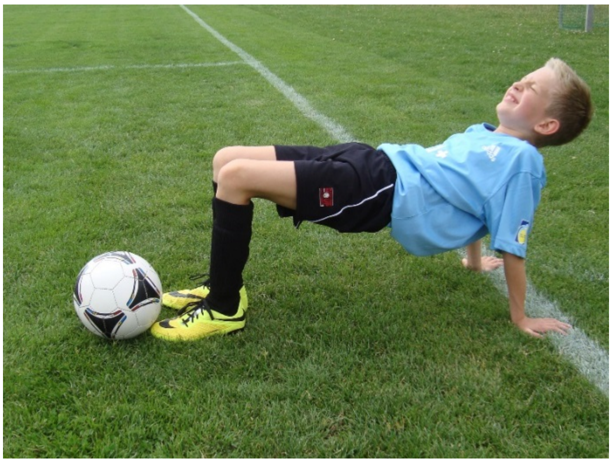

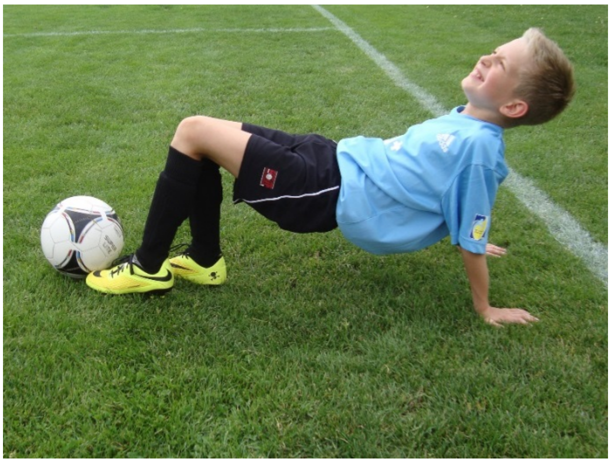

Level 5: Kugelläufer

Ausgangsposition: Wie Level 1, jedoch stützen sich die Spieler mit den Füssen auf dem Ball ab.

Aktion: Die Spieler rollen den Ball unter den Füssen und bewegen sich dadurch langsam und kontrolliert vorwärts.

Wiederholungen: 3 Durchgänge (je nach Leistungsniveau 3-7 m).

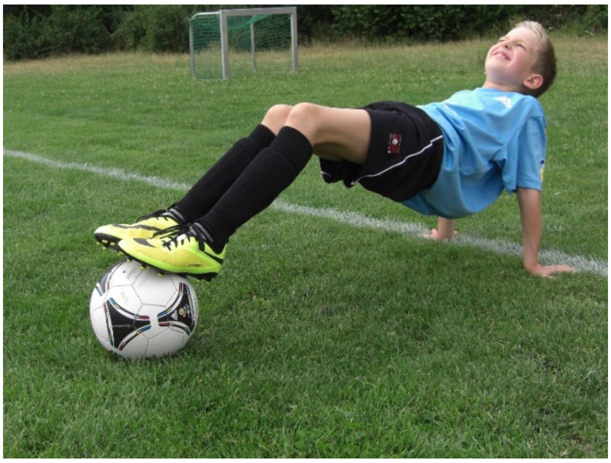

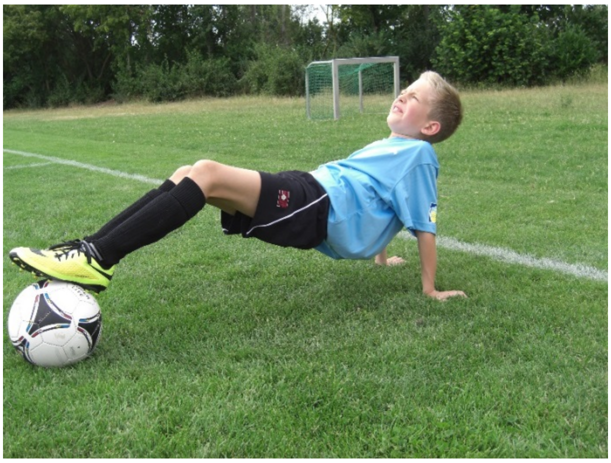

Übung 6: Spiderman

Richtig und wichtig:

- ✓ Das Gesäss soll oben gehalten werden.
- ✓ Der Körper ist in einer möglichst geraden Linie von den Schultern bis zu den Knien.
- ✓ Der Kopf ist in neutraler Stellung.
- ✓ Die Füsse sind immer *unter* oder *vor* den Knien (Kniewinkel immer grösser als 90°)

Diese Fehler bitte unbedingt korrigieren:

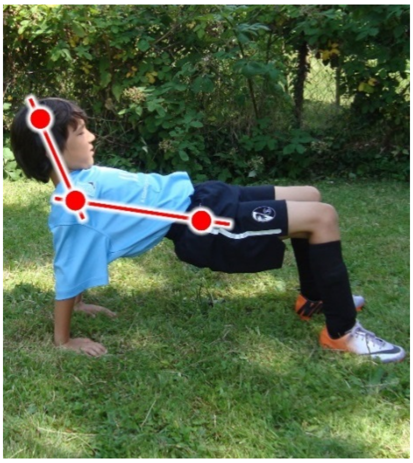

Fehler: Falsche Kopfposition

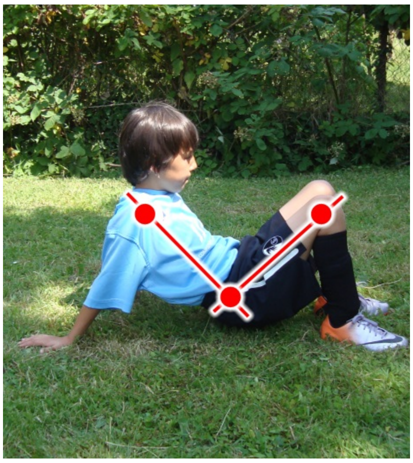

Fehler: Durchhängen des Körpers

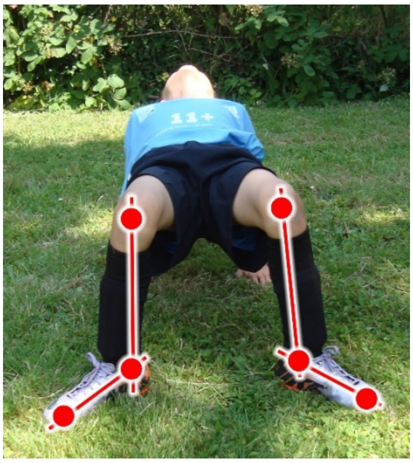

Fehler: Ausdrehen der Füsse

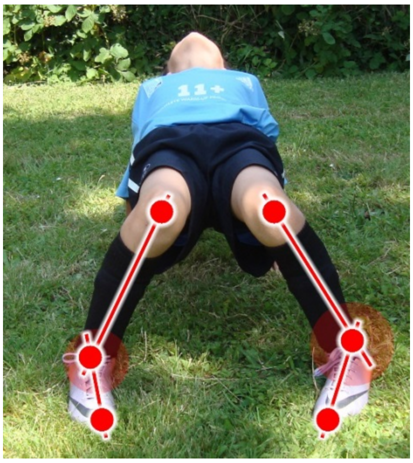

Fehler: Eindrehen der Füsse

Übung 7: Seitliches Abrollen

Für alle Übungen gilt:

Fokus: Fallen und Abrollen lernen.

Ziel: Die Rollübungen in beide Richtungen zu beherrschen.

Anweisung an die Spieler: „Macht euch beim Abrollen klein und rund wie ein Ball!"

Level 1: Aus der Hocke

Ausgangsposition: Die Spieler sind in der Hockposition mit mindestens 2 m Abstand zu den Mitspielern.

Aktion: Die Spieler setzen beide Hände vor dem Knie auf dem Boden auf. Nun senken die Spieler den Kopf, sodass das Kinn nahe an der Brust ist und rollen sich diagonal über Arm, Schulter und Rücken ab. Beispiel: Soll über die rechte Seite abgerollt werden, so ist der rechte Arm vorne in einer gebeugten Position. Die Abrollbewegung erfolgt dann über die Aussenseite des rechten Armes, die rechte Schulter und schliesslich diagonal über den Rücken zurück auf die Füsse. Der Trainer wartet nach jeder Rolle mindestens 5 Sekunden bis zum nächsten Kommando. Die Spieler konzentrieren sich auf jede einzelne Rolle. Der Trainer gibt für jede Rolle die Richtung an.

Wiederholungen: 5 Rollen pro Seite.

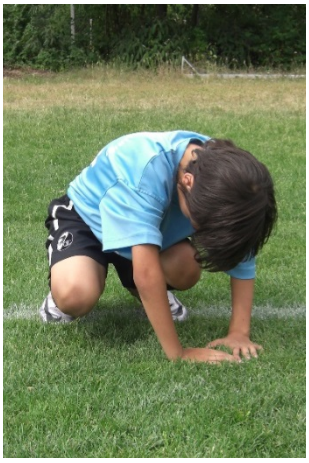

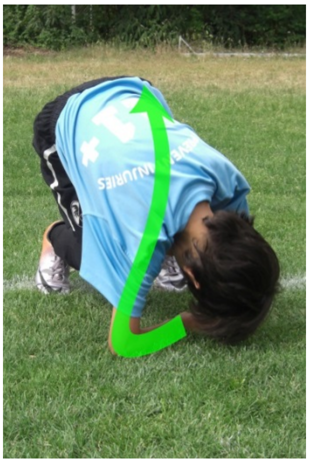

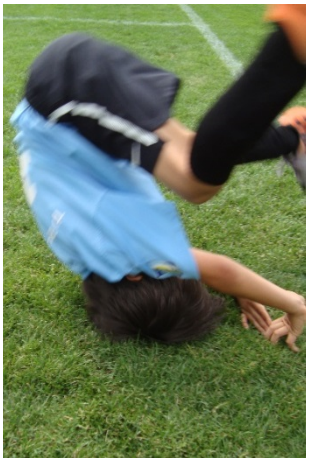

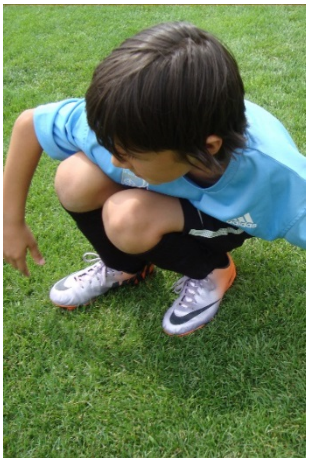

Übung 7: Seitliches Abrollen

Level 2: Langsam aus dem Stand

Ausgangsposition: Die Spieler sind im aufrechten Stand mit mindestens 2 m Abstand zu den Mitspielern.

Aktion: Das in Level 1 erlernte Abrollen wird nun aus dem aufrechten Stand eingeleitet. Die Spieler gehen aus Stand in die Hocke, indem sie die Knie beugen. Gleichzeitig richten sie den Blick nach unten und bewegen das Kinn zur Brust. Der Handaufsatz erfolgt langsam und kontrolliert, sodass die Spieler den Ablauf verinnerlichen können. Die anschliessende Rollbewegung entspricht Level 1.

Wiederholungen: 5 Rollen pro Seite.

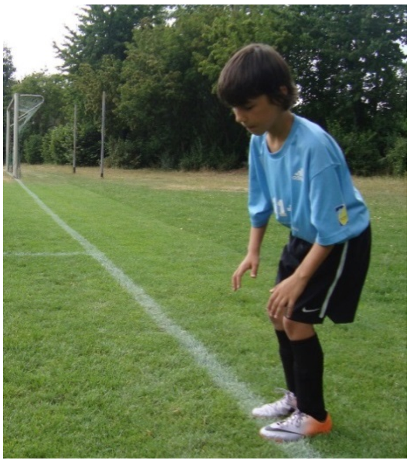

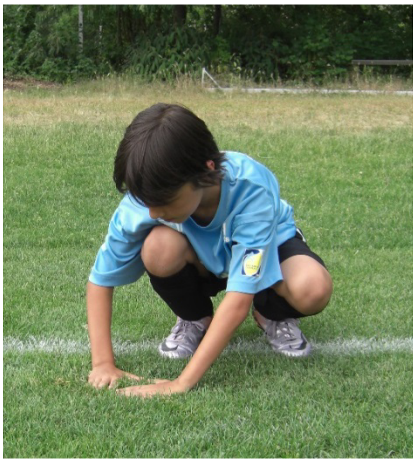

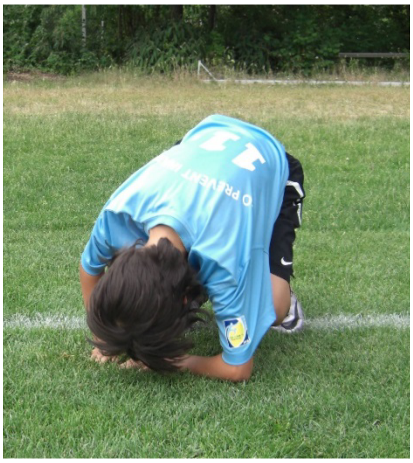

Level 3: Dynamisch aus dem Stand

Ausgangsposition: Wie Level 2.

Aktion: Das in Level 2 erlernte „in-die Hocke-gehen" wird nun schneller und dynamischer ausgeführt und die in Level 1 erlernte Abrollbewegung wird dynamisch eingeleitet.

Wiederholungen: 5 Rollen pro Seite.

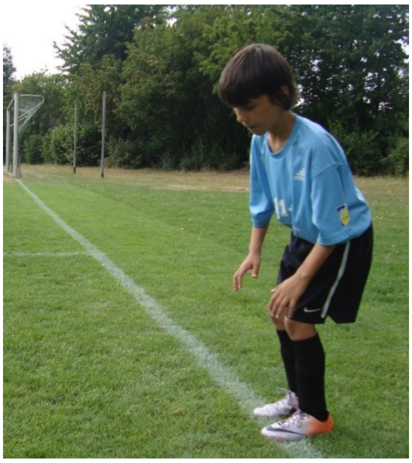

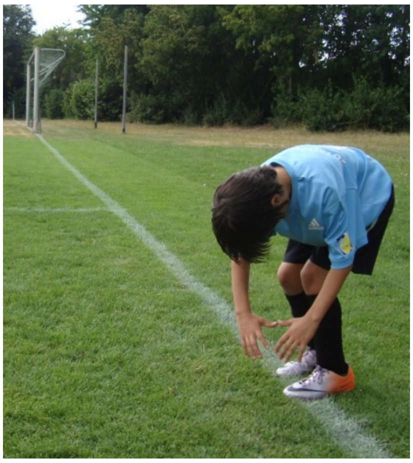

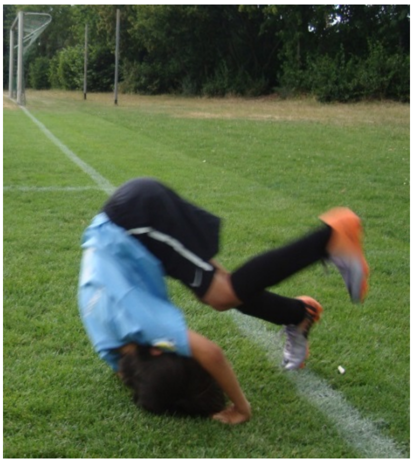

Übung 7: Seitliches Abrollen

Level 4: Aus dem langsamen Gehen

Ausgangsposition: Wichtig ist, dass die Spieler das in Level 3 erlernte schnelle Abrollen aus dem Stand in beide Richtungen sicher ausführen können.

Aktion: Das in Level 3 erlernte in die Hocke gehen und Abrollen wird aus dem langsamen Gehen eingeleitet.

Wiederholungen: 5 Rollen pro Seite.

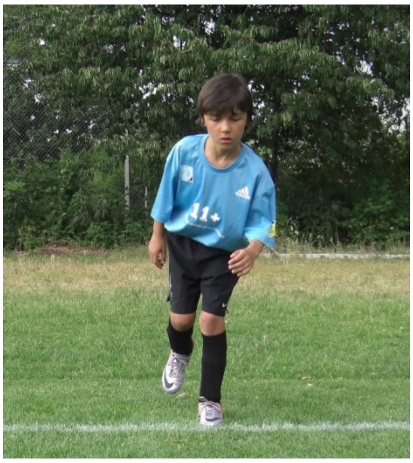

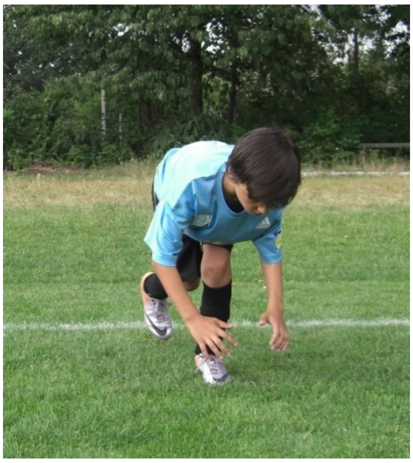

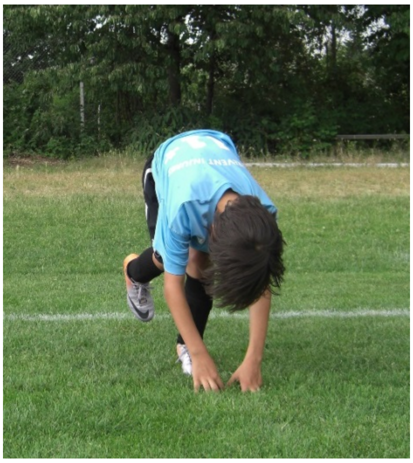

Level 5: Aus der schnelleren Vorwärtsbewegung

Ausgangsposition: Wichtig ist, dass die Spieler die in Level 4 erlernte Bewegung sicher in beide Richtungen ausführen können. Erst dann darf Level 5 ausgeführt werden.

Aktion: Wie Level 4, jedoch aus der schnelleren Vorwärtsbewegung (schnelles Gehen/Joggen) heraus.

Wiederholungen: 5 Rollen pro Seite.

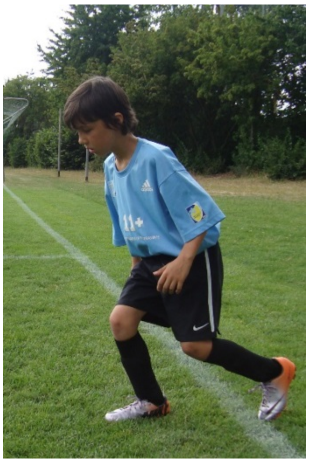

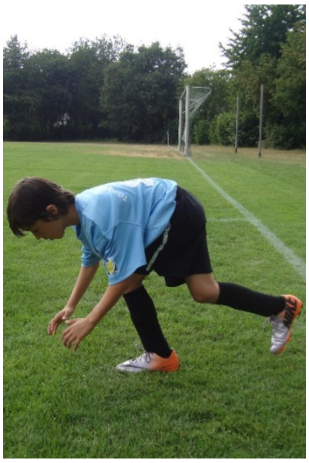

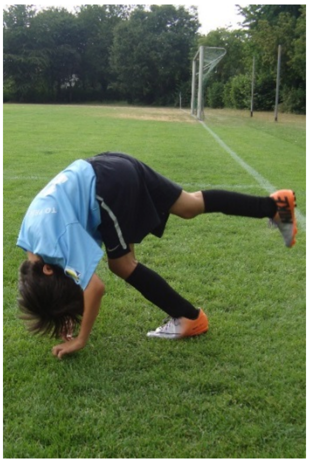

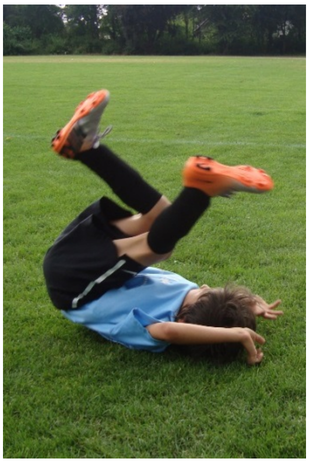

Übung 7: Seitliches Abrollen

Wichtig und richtig:

- ✓ Das Kinn wird zur Brust genommen.
- ✓ Über die Aussenseite des Arms, die Schulter und schliesslich diagonal über den Rücken abrollen!
- ➢ Vor jeder Rolle unbedingt auf genügend Abstand zu den anderen Spielern achten!
- ➢ Ausreichende Pause nach jeder Rolle (mindestens 5 Sekunden)
- ➢ Der Trainer gibt ein Startkommando und die Richtung für jede Rolle vor

Diese Fehler bitte unbedingt korrigieren:

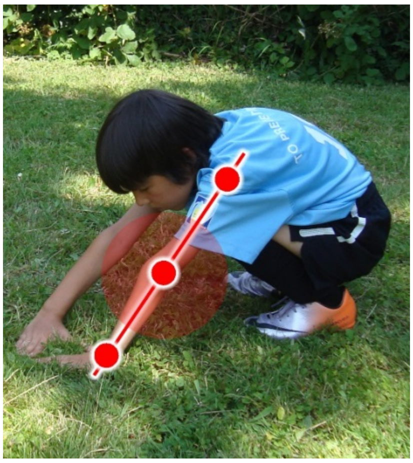

Fehler: Arme sind gestreckt

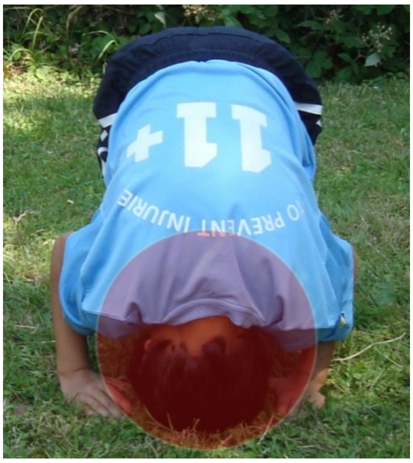

Fehler: Kopf berührt den Boden, gerades Rollen über den Rücken

Gestaltung und Fotos

Roland Rössler, Oliver Faude, Eric Lichtenstein

Kontakt

Roland Rössler, M. Sc.

Universität Basel

Departement für Sport, Bewegung und Gesundheit (DSBG)

Postadresse: Birsstrasse 320B, CH - 4052 Basel

Tel. 0041 (0)61 377 87 39

E-Mail: kinderfussball-dsbg@unibas.ch

Copyright

Fédération Internationale de Football Association

FIFA-Strasse 20

P.O. Box

8044 Zurich

Switzerland

F-MARC FOOTBALL FOR HEALTH FIFA © 2007 FIFA TM

DSBG UNIVERSITY OF BASEL Department of Sport, Exercise and Health
