# Supplementary material for: A Multinational Cluster Randomised Controlled Trial to Assess the Efficacy of ‘11+ Kids’: A Warm-Up Programme to Prevent Injuries in Children’s Football
Source: Sports Med. 2017 Dec 22;48(6):1493–504. doi: 10.1007/s40279-017-0834-8 (PMC5948238; doi:10.1007/s40279-017-0834-8)
Supplement: Supplementary file 2 — ‘11+ Kids’ short version of the manual (study version) [file 40279_2017_834_MOESM2_ESM.pdf]

Sports Medicine ISSN: 0112-1642 (Print) 1179-2035 (Online) Springer International Publishing

A Multinational Cluster Randomised Controlled Trial to Assess the Efficacy of '11+ Kids': A Warm-Up Programme to Prevent Injuries in Children's Football

Short title: Injury prevention in children's football

Roland Rössler[1,2*] (RR, postdoc), Astrid Junge[3,4,5] (AJ, professor), Mario Bizzini[3,4] (MB, physiotherapist), Evert Verhagen[2] (EV, assistant professor), Jiri Chomiak[6] (JC, medical doctor), Karen aus der Fünten[7] (KadF, medical doctor) Tim Meyer[7], (TM, professor), Jiri Dvorak[3,4] (JD, professor), Eric Lichtenstein[1] (EL, research assistant), Florian Beaudouin[7] (FB, research assistant), Oliver Faude[1] (OF, senior researcher)

1 Department of Sport, Exercise and Health University of Basel, Basel Switzerland

2 Amsterdam Collaboration on Health & Safety in Sports and Department of Public and Occupational Health, Amsterdam Movement Science VU University Medical Center, Amsterdam Netherlands

3 Swiss Concussion Center, Zurich Switzerland

4 Schulthess Clinic, Zurich Switzerland

5 Medical School Hamburg, Hamburg Germany

6 Orthopaedic Department 1st Faculty of Medicine Charles University and IPVZ and Hospital Na Bulovce and FIFA med. Centre, Prague Czech Republic

7 Institute of Sports and Preventive Medicine Saarland University, Saarbrücken Germany

* Corresponding author

Roland Rössler, PhD

University of Basel

Department of Sport, Exercise and Health

Birsstrasse 320B

4052 Basel Switzerland

roland.roessler@unibas.ch

ORCID: 0000-0002-6763-0694

FIFA 11+ Kids: Ein Aufwärmprogramm zur Verletzungsprävention im Kinderfussball

Die Kurzversion „für den Platz“

	Level 1	Level 2	Level 3	Level 4	Level 5
**Übung 1: Laufspiel „Wachmann“**	**Stopp-Kommando hören**<br>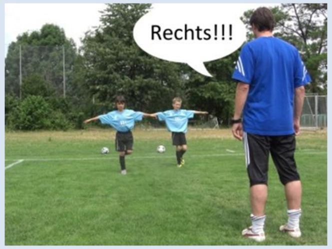	**Stopp-Kommando sehen**<br>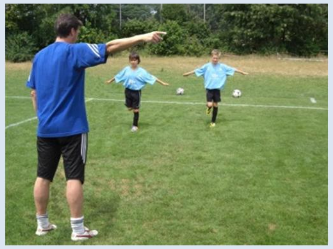	**Ball in den Händen und Kommando hören**<br>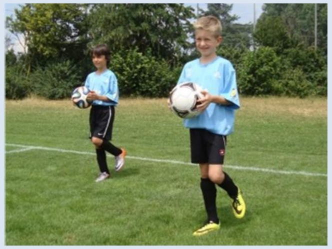	**Ball in den Händen und Kommando sehen**<br>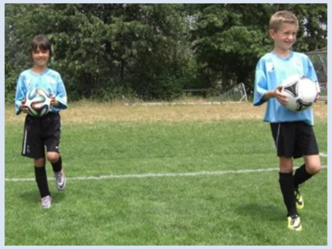	**Ball dribbeln und Kommando hören**<br>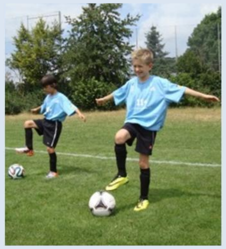
**Übung 2: Skating-Sprünge**	**Landen lernen**<br>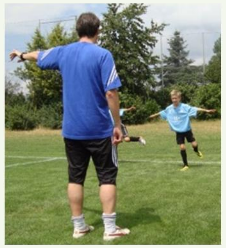	**Ball in beiden Händen**<br>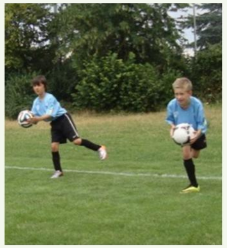	**Ball in einer Hand balancieren**<br>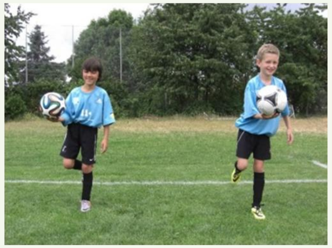	**Ball auf den Boden tippen**<br>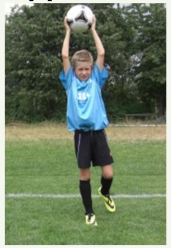 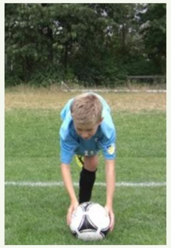	**Dynamische Standwaage mit Ball**<br>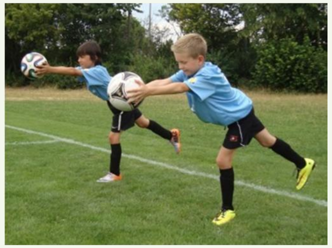
**Übung 3: Einbeinstand**	**Ball zuwerfen**<br>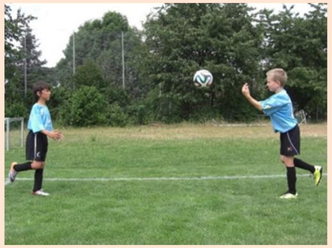	**Ball zuwerfen und um das Spielbein kreisen**<br>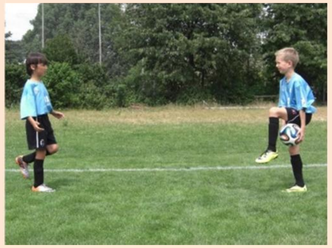	**Passspiel**<br>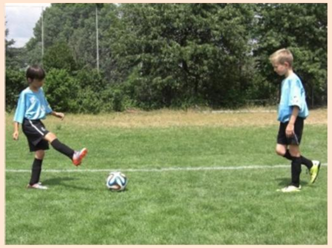	**Ball zuwerfen und mit dem Fuss zurückpassen**<br>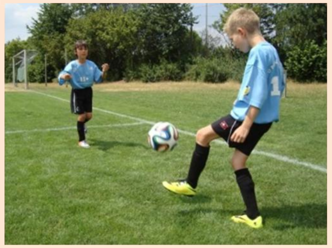	**Gleichgewicht des Partners testen**<br>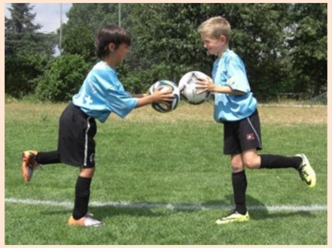

	Level 1	Level 2	Level 3	Level 4	Level 5
Übung 4:<br>Liegestütz	Tunnel<br>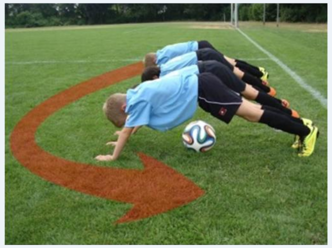	Unterarmstütz: Schien-beine auf dem Ball<br>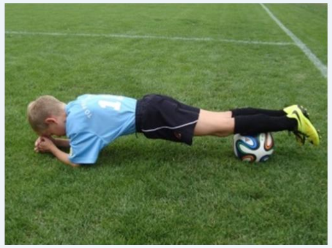	Ball um die Hände rollen<br>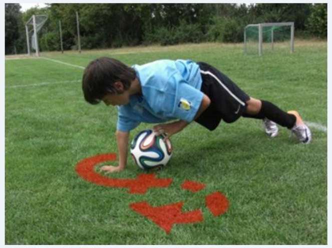	Ball zwischen Händen und Füssen rollen<br>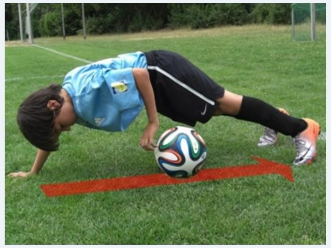	Hände auf dem Ball<br>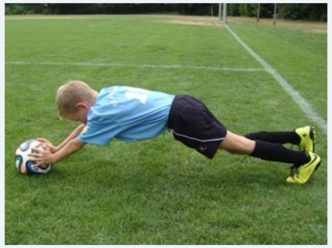
Übung 5:<br>Einbeinsprünge	Nach vorne<br>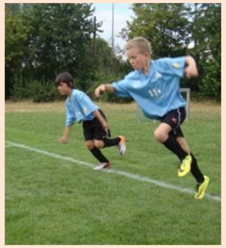	Vor und zurück<br>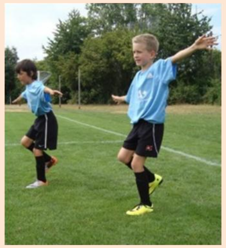	Seitwärts<br>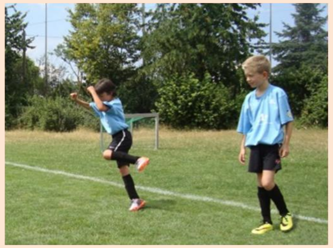	Trainer gibt die Richtung an<br>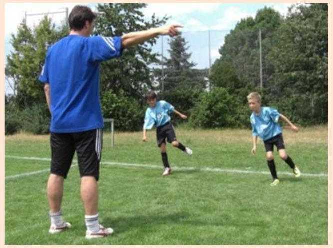	Trainer gibt Richtung an Ball in den Händen<br>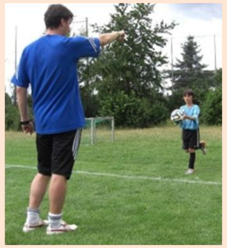
Übung 6:<br>Spiderman	Ball antippen<br>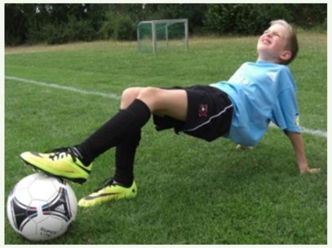	Recken und Strecken<br>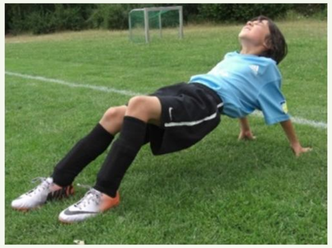	Krabbeln<br>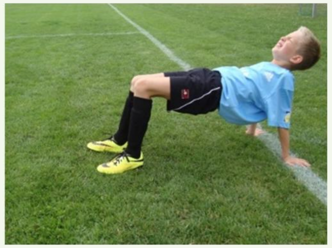	Dribbeln<br>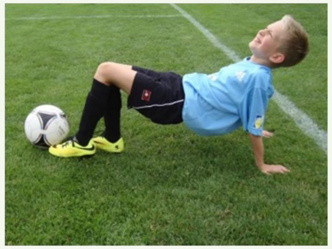	Kugelläufer<br>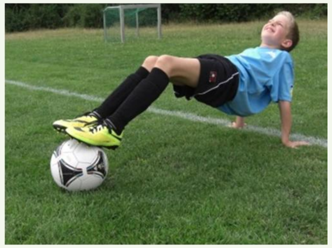
Übung 7:<br>Seitliches<br>Abrollen	Aus der Hocke<br>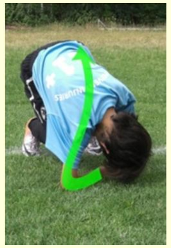	Langsam aus dem Stand<br>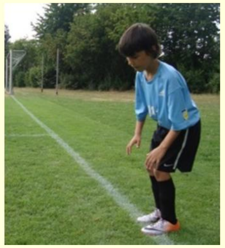	Dynamisch aus dem Stand<br>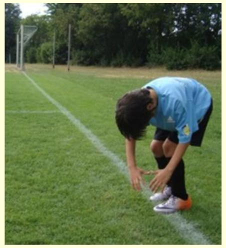	Aus dem langsamen Gehen<br>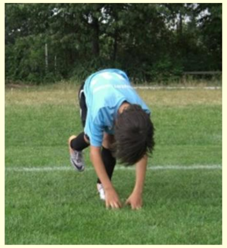	Aus der schnelleren Vorwärtsbewegung<br>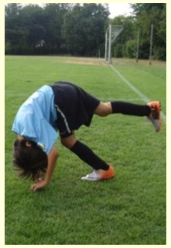
