# Supplementary material for: A Multinational Cluster Randomised Controlled Trial to Assess the Efficacy of ‘11+ Kids’: A Warm-Up Programme to Prevent Injuries in Children’s Football
Source: Sports Med. 2017 Dec 22;48(6):1493–504. doi: 10.1007/s40279-017-0834-8 (PMC5948238; doi:10.1007/s40279-017-0834-8)
Supplement: Supplementary file 3 — Table 1: General evaluation of ‘11+ Kids’ by coaches of the intervention group at the end of the season. For items 1–11, a five-level Likert scale was used as follows: 1: Strongly disagree, 2: Disagree, 3: Neither agree nor disagree, 4: Agree, 5: Strongly agree. Table 2: Exercise-specific evaluation of ‘11+ Kids’ by coaches of the intervention group at the end of the season. For items 1–3, a five-level Likert scale was used as follows: 1: Strongly disagree, 2: Disagree, 3: Neither agree nor disagree, 4: Agree, 5: Strongly agree [file 40279_2017_834_MOESM3_ESM.pdf]

Sports Medicine ISSN: 0112-1642 (Print) 1179-2035 (Online) Springer International Publishing

A Multinational Cluster Randomised Controlled Trial to Assess the Efficacy of '11+ Kids': A Warm-Up Programme to Prevent Injuries in Children's Football

Short title: Injury prevention in children's football

Roland Rössler[1,2*] (RR, postdoc), Astrid Junge[3,4,5] (AJ, professor), Mario Bizzini[3,4] (MB, physiotherapist), Evert Verhagen[2] (EV, assistant professor), Jiri Chomiak[6] (JC, medical doctor), Karen aus der Fünten[7] (KadF, medical doctor) Tim Meyer[7], (TM, professor), Jiri Dvorak[3,4] (JD, professor), Eric Lichtenstein[1] (EL, research assistant), Florian Beaudouin[7] (FB, research assistant), Oliver Faude[1] (OF, senior researcher)

1 Department of Sport, Exercise and Health University of Basel, Basel Switzerland

2 Amsterdam Collaboration on Health & Safety in Sports and Department of Public and Occupational Health, Amsterdam Movement Science VU University Medical Center, Amsterdam Netherlands

3 Swiss Concussion Center, Zurich Switzerland

4 Schulthess Clinic, Zurich Switzerland

5 Medical School Hamburg, Hamburg Germany

6 Orthopaedic Department 1st Faculty of Medicine Charles University and IPVZ and Hospital Na Bulovce and FIFA med. Centre, Prague Czech Republic

7 Institute of Sports and Preventive Medicine Saarland University, Saarbrücken Germany

* Corresponding author

Roland Rössler, PhD

University of Basel

Department of Sport, Exercise and Health

Birsstrasse 320B

4052 Basel Switzerland

roland.roessler@unibas.ch

ORCID: 0000-0002-6763-0694

Electronic supplementary material 3: General evaluation of ‘11+ Kids’ by coaches of the intervention group at the end of the season. For items 1 to 11 a five-level Likert scale was used as follows: 1: Strongly disagree, 2: Disagree, 3: Neither agree nor disagree, 4: Agree, 5: Strongly agree.

	N	Min	Max	Mean	SD
**After the briefing by the study assistant I was able to instruct the players independently.**	128	1	5	4.71	0.74
**The printed ‘11+ Kids’ manual is helpful.**	128	2	5	4.77	0.62
**The time requirement is reasonable.**	128	1	5	3.37	1.20
**‘11+ Kids’ can be integrated into the training routine without problems.**	128	2	5	3.92	1.01
**‘11+ Kids’ can be performed in bad weather conditions without problems.**	126	1	5	3.60	1.07
**‘11+ Kids’ can be performed indoors without problems.**	126	1	5	4.42	0.96
**After the winter break the players were able to proceed on the same level.**	124	2	5	4.10	0.90
**I believe that the programme can prevent injuries.**	126	1	5	3.90	0.92
**I believe that the programme can improve player's performance.**	124	1	5	3.49	0.90
**After the programme the players were sufficiently warmed-up to begin with the training.**	126	1	5	3.93	0.96
**In general, I find injury prevention important.**	122	3	5	4.80	0.52
**How long did it take to perform the programme on average? [min]**	124	5	35	18.05	4.40
**Did you do the programme in every training session? [%]**	122			36.8	

Electronic supplementary material 4: Exercise-specific evaluation of '11+ Kids' by coaches of the intervention group at the end of the season. For items 1 to 3 a five-level Likert scale was used as follows: 1: Strongly disagree, 2: Disagree, 3: Neither agree nor disagree, 4: Agree, 5: Strongly agree.

**Exercise**	**Running game**		**Skating jumps**		**Ball passing**		**Push-up**		**Single leg jumps**		**Spiderman**		**Falling techniques**	
	**Mean**	**SD**	**Mean**	**SD**	**Mean**	**SD**	**Mean**	**SD**	**Mean**	**SD**	**Mean**	**SD**	**Mean**	**SD**
**The exercise was fun for the players.**	3.82	0.86	3.78	0.86	4.18	0.73	4.08	0.97	3.88	0.99	3.96	1.02	3.82	1.20
**Graduation of the levels is reasonable.**	4.14	0.81	4.28	0.66	4.30	0.81	4.27	0.78	4.28	0.82	4.11	0.84	4.07	0.86
**Feasibility of the exercise is high.**	4.43	0.72	4.24	0.83	4.37	0.71	4.03	0.86	4.44	0.65	3.87	1.04	3.48	1.15
**Level (1-5) performed at the end of the season:**	4.36	0.77	4.13	0.89	4.17	0.76	3.79	1.01	4.29	0.69	3.74	0.84	3.63	1.16
**I did modifications to the exercise. [%]**	9.7		2.7		2.7		12.0		6.5		12.0		5.6	
